# Establishment of a Patient-Derived, Magnetic Levitation-Based, Three-Dimensional Spheroid Granuloma Model for Human Tuberculosis

**DOI:** 10.1128/mSphere.00552-21

**Published:** 2021-07-21

**Authors:** Leigh A. Kotze, Caroline G. G. Beltran, Dirk Lang, Andre G. Loxton, Susan Cooper, Maynard Meiring, Coenraad F. N. Koegelenberg, Brian W. Allwood, Stephanus T. Malherbe, Andriette M. Hiemstra, Brigitte Glanzmann, Craig Kinnear, Gerhard Walzl, Nelita du Plessis

**Affiliations:** a DST-NRF Centre of Excellence for Biomedical Tuberculosis Research, South African Medical Research Council Centre for Tuberculosis Research, Division of Molecular Biology and Human Genetics, Faculty of Medicine and Health Sciences, Stellenbosch University, Cape Town, South Africa; b Confocal and Light Microscopy Imaging Facility, University of Cape Town, Cape Town, South Africa; c South African Medical Research Council Genomics Centre, Cape Town, South Africa; d Division of Pulmonology, Department of Medicine, Stellenbosch University and Tygerberg Academic Hospital, Cape Town, South Africa; University of Kentucky

**Keywords:** 3D cell culture, granuloma, spheroid, tuberculosis

## Abstract

Tuberculous granulomas that develop in response to Mycobacterium tuberculosis (M. tuberculosis) infection are highly dynamic entities shaped by the host immune response and disease kinetics. Within this microenvironment, immune cell recruitment, polarization, and activation are driven not only by coexisting cell types and multicellular interactions but also by M. tuberculosis-mediated changes involving metabolic heterogeneity, epigenetic reprogramming, and rewiring of the transcriptional landscape of host cells. There is an increased appreciation of the *in vivo* complexity, versatility, and heterogeneity of the cellular compartment that constitutes the tuberculosis (TB) granuloma and the difficulty in translating findings from animal models to human disease. Here, we describe a novel biomimetic *in vitro* three-dimensional (3D) human lung spheroid granuloma model, resembling early “innate” and “adaptive” stages of the TB granuloma spectrum, and present results of histological architecture, host transcriptional characterization, mycobacteriological features, cytokine profiles, and spatial distribution of key immune cells. A range of manipulations of immune cell populations in these spheroid granulomas will allow the study of host/pathogen pathways involved in the outcome of infection, as well as pharmacological interventions.

**IMPORTANCE** TB is a highly infectious disease, with granulomas as its hallmark. Granulomas play an important role in the control of M. tuberculosis infection and as such are crucial indicators for our understanding of host resistance to TB. Correlates of risk and protection to M. tuberculosis are still elusive, and the granuloma provides the perfect environment in which to study the immune response to infection and broaden our understanding thereof; however, human granulomas are difficult to obtain, and animal models are costly and do not always faithfully mimic human immunity. In fact, most TB research is conducted *in vitro* on immortalized or primary immune cells and cultured in two dimensions on flat, rigid plastic, which does not reflect *in vivo* characteristics. We have therefore conceived a 3D, human *in vitro* spheroid granuloma model which allows researchers to study features of granuloma-forming diseases in a 3D structural environment resembling *in vivo* granuloma architecture and cellular orientation.

## INTRODUCTION

A hallmark of tuberculosis (TB) is the formation of granulomatous lesions in response to M. tuberculosis-infected phagocytes, such as alveolar macrophages (AM), within the pulmonary space, inducing a chronic inflammatory response. Additional macrophages are recruited to the site of infection to form the “core” structure of the granuloma, characterized as an innate response to infection. Recent literature shows that early M. tuberculosis infection occurs almost exclusively in airway-resident alveolar macrophages, whereafter M. tuberculosis-infected, but not uninfected, alveolar macrophages localize to the lung interstitium, preceding M. tuberculosis uptake by recruited monocyte-derived macrophages and neutrophils (1). Other immune cell types such as interstitial macrophages, monocytes, dendritic cells, neutrophils, and lymphocytes (T and B cells) are recruited to the site of infection, where they are collected and organized around the core to form mature granuloma structures to contribute to an adaptive immune response (2–4). Granulomas are thus the main site of the host-pathogen interaction, primarily aimed at preventing M. tuberculosis dissemination, but during immune dysregulation, they can also function as a niche for M. tuberculosis survival and persistence (5).

During TB disease, the damage to host lung tissue, as evidenced by necrosis and cavitation, is associated with bacterial persistence and the development of drug-resistant M. tuberculosis. Ultimately, TB patients harbor a range of granulomas, which may comprise a spectrum of solid nonnecrotizing, necrotic, and caseous granulomas, each with its own distinct microenvironment; these have been demonstrated to not be limited to active TB patients but are also observed in animals/nonhuman primates with both latent and reactivation cases of TB (6). The mere formation of granulomas is thus insufficient for infection control and instead must function with the appropriate combination of host control measures, for example, the correct balance of pro- and anti-inflammatory response mediators. The outcome of the host response to infection may be beneficial in that some granulomas resolve completely (sterilizing cure), while others progress to caseation and rupture, allowing for the uncontrolled dissemination of M. tuberculosis into the surrounding tissue; both scenarios have been known to occur within the same individual (7). The heterogeneity of granulomas even within the same patient highlights the need to study each of these individual structures as a whole to assess host-pathogen interactions at an individual granuloma level. Progress in understanding M. tuberculosis pathogenesis at a patient granuloma level has been poor due to the difficulty in obtaining fresh human TB granulomatous lung tissue. For this reason, patient-driven immune responses to M. tuberculosis have been assessed mainly in primary host immune cells, cultured *in vitro* as traditional cell cultures on plastic plates optimized for tissue culture. Such traditional cell cultures with uniform exposure to pathogen/immune mediators have served as important tools for studying host-M. tuberculosis interactions. However, these nonphysiological conditions fail to recapitulate key elements of cells residing in the complex tissue microenvironment, including cell concentration, cell types, cell motility, multicellular interactions, cell expansion, spatiotemporal kinetics, and geometries (8, 9). Such discrepancies present a significant barrier to interpretation and translation of findings from basic science and vaccine immunogenicity, drug efficacy, and prevention-of-infection studies, ultimately limiting the impact of TB research on human health.

While animal models have contributed significantly to our understanding of the mammalian immune system, numerous examples have shown that laboratory animal species do not faithfully, or in full, mimic human immunity or TB disease (10–12). To overcome these obstacles, successful three-dimensional (3D) *in vitro* models can contribute to the ethos of the 3Rs of animal research (replacement, reduction, and refinement) by replacing the use of animals in research with insentient material obtained from willing donors, thereby also promoting human relevance (13). Advanced *in vitro* models (derived from *in vivo* spatial information) that recapitulate the spatial interactions between recruited monocytes, T cells, and B cells in the context of the human lung granuloma are lacking but are incredibly necessary for the future of TB research and the successful dissection of disease pathology (14).

In this study, we utilized primary human alveolar macrophages retrieved from the site of disease and autologous adaptive immune cells isolated from the periphery to successfully establish an *in vitro* 3D spheroid granuloma model of human TB using magnetic cell levitation and BCG infection. Magnetic cell levitation is a recently developed method used to generate tumor spheroids during which tumor cells are preloaded with magnetic nanospheres which electrostatically attach to cell membranes to form multicellular spheroids suspended in culture via an external magnetic field. Nanospheres subsequently detach from the cells, allowing unsupported growth as the structure starts to mimic extracellular matrix (ECM) conditions (15). Here, we describe an *in vitro* model which could be used to examine stages of granuloma formation, demonstrated here as early (innate) and late (adaptive) granuloma types. These complex multidimensional structures are investigated at the morphological level and subsequently dissociated to assess single-cell characteristics for comparison to traditional cell cultures prepared using identical cell types, ratios, and culturing conditions. We demonstrate, along with the method of construction, the possible applications of this model, including a wide range of immunological assays. This model has proven to be stable and reliable for the investigation of mycobacterial granulomas in a tissue culture setting, opening the door for expansion of this model into additional molecular biology avenues.

## RESULTS

The results of this proof-of-concept study demonstrate that the human *in vitro* 3D spheroid granuloma model is capable of being successfully interrogated using multiple molecular biology platforms.

### Macroscopic and microscopic differences in granuloma structural formation exist due to cell composition and mycobacterial infection.

**FIG 1:**
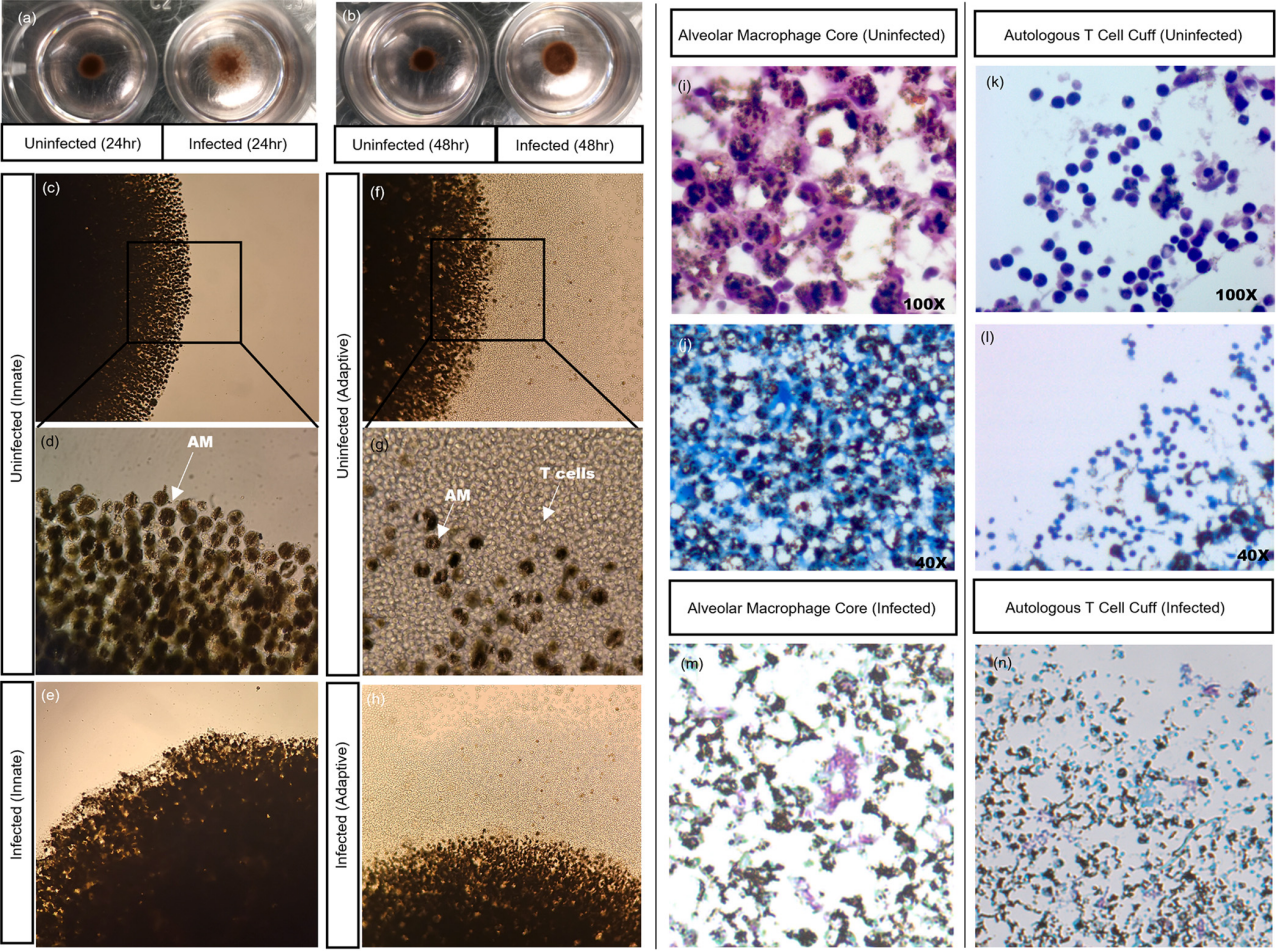
Visual differences in cellular organization between uninfected and BCG-infected granuloma structures can be observed after (a) 24 and (b) 48 h of magnetic levitation, with BCG-infected structures displaying less robust structural integrity (the magnetic levitation drive was removed briefly for these images to be taken). Differences in cellular organization could also be visualized using light microscopy, with uninfected innate granuloma structures at (c) lower (20×) and (d) higher (40×) magnifications and (e) BCG-infected innate granuloma structures displaying a clear lack of a lymphocytic cuff at the end of the culture period. Both the uninfected adaptive granuloma structures at (f) lower (20×) and (g) higher (40×) magnifications and (h) BCG-infected adaptive granuloma structures displayed the presence of a lymphocytic cuff (unlabeled, clear cells) surrounding the NanoShuttle-labeled AM core (darker cells) at the end of the culture period, during which time both magnetic levitation and magnetic bioprinting were used. These images were taken using an inverted light microscope. Light microscopy can also be used to investigate various staining methods. Using the H&E staining method, we corroborated our findings from the inverted microscope demonstrating (i) the alveolar macrophage core and (k) the CD3^+^ autologous T cell cuff. ZN staining of the sections demonstrated the uninfected nature of the structures, in both (j) the core and (l) the cuff, while ZN staining of infected sections demonstrated acid-fast bacilli both (m) in the core and (n) near the cuff of the structure.

NanoShuttle-labeled alveolar macrophages were levitated for 48 h at the beginning of the model construction, and granuloma cores were observed as hanging cultures with spheroids suspended just underneath the surface of the culture media. After the first 24 h of levitation, the magnetic levitation drive was removed briefly to investigate the structural integrity of the granulomas without magnetic influence. The assembly of the alveolar macrophage (AM) core could already be visualized at the 24-h time point with BCG-infected cores appearing less “stable” than uninfected cores (Fig. 1a), with this apparent instability improving by the 48-h culture time point (Fig. 1b). At the end of the culture period, we could see macroscopic variations in the morphology of uninfected (Fig. 1c and d) and BCG-infected (Fig. 1e) innate granulomas as well as between the uninfected (Fig. 1f and g) and BCG-infected (Fig. 1h) adaptive granulomas. A “cuff” of unlabeled autologous CD3^+^ T cells was clearly seen to be surrounding the darker, NanoShuttle-labeled AM core (Fig. 1f and h), compared to the innate core lacking the cuff due to the lack of autologous CD3^+^ T cells (Fig. 1c and e). While these visual differences are important for defining structural phenotypes during the early stages of this model’s development, information regarding details like the infiltration of autologous CD3^+^ T cells into the AM core could not be inferred without immunofluorescent microscopic assessment.

Granuloma structures were further investigated at the cellular level to interrogate the composition in a more detailed manner. For the uninfected granulomas, both H&E (Fig. 1i) and Ziehl-Neelsen (ZN) (Fig. 1j) stains demonstrated that no acid-fast bacilli are present within the AM core. The adaptive granuloma structures demonstrated a cuff of autologous lymphocytes, based on known morphology, as seen using both H&E (Fig. 1k) and ZN (Fig. 1l) staining. ZN staining of an infected granuloma structure demonstrated acid-fast bacilli both in the core of the structure (Fig. 1m) and near the cuff (Fig. 1n). It is interesting to note that the cores of the adaptive granuloma structures were dominantly macrophages (Fig. 1i), suggesting minimal infiltration by autologous CD3^+^ T cells from the cuff at this infection time point. Inferences could suggest that the architecture of granulomas with noninfiltrating lymphocytes encapsulating the AM core represent a structural component of mycobacterial infection control/failure (16–18); alternatively, this could also be a methodological limitation whereby the CD3^+^ T cells have simply not had sufficient time to migrate through the core.

### Magnetic levitation combined with bioprinting successfully produces a morphologically and physiologically relevant 3D TB lung granuloma.

**FIG 2:**
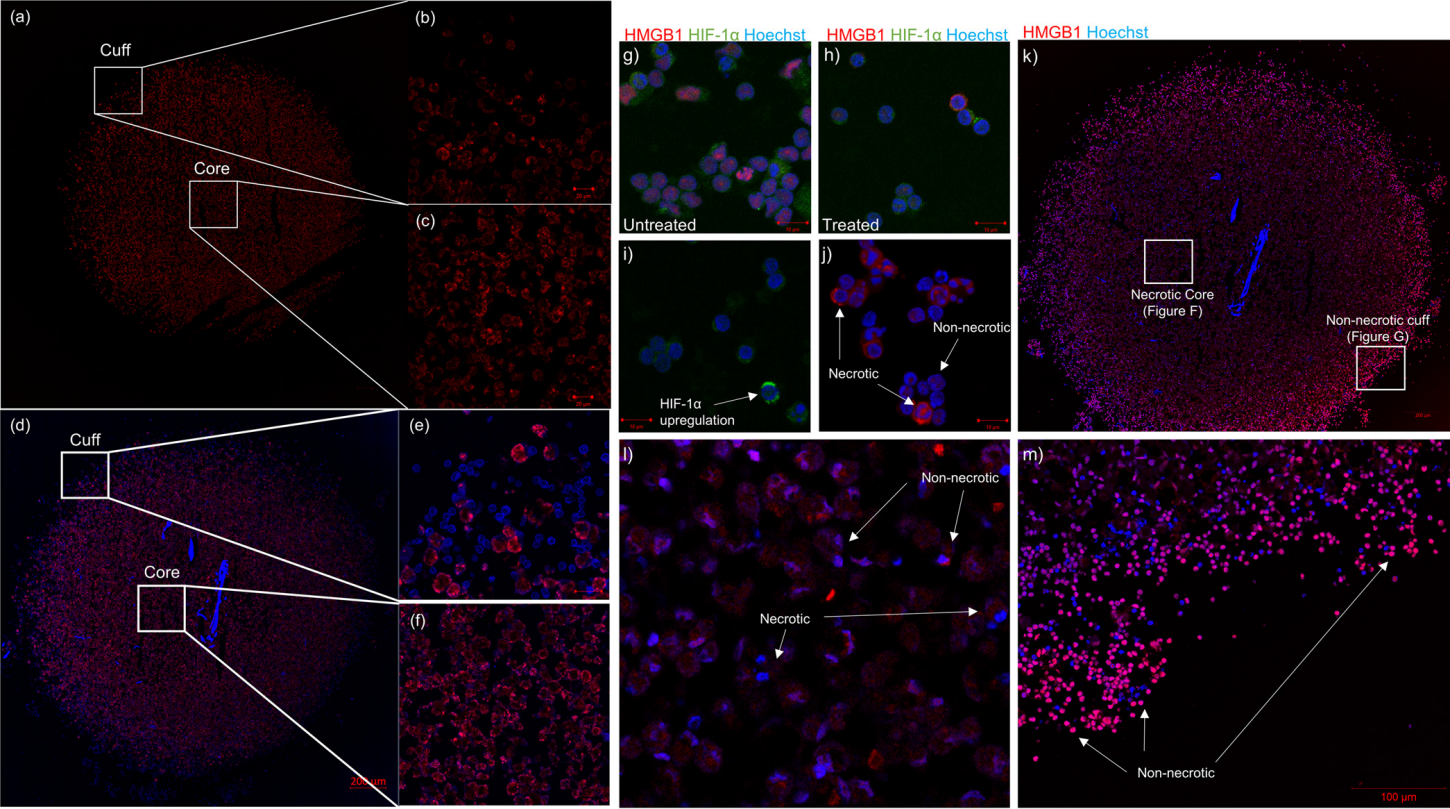
Tile scan (14 by 14) of a 3D uninfected innate granuloma structure depicting (a) the entire structure, (b) the “cuff” devoid of autologous CD3^+^ T cells, and (c) the AM-dominant core. AM were stained with CD206 PE-CF594 (red). Nuclei were left unstained. A tile scan (18 by 18) of a 3D uninfected adaptive granuloma structure depicting (d) the entire structure, (e) the autologous CD3^+^ T cell-dominant cuff, and (f) the AM-dominant core. AM were stained with CD206 PE-CF594 (red), and autologous CD3^+^ T cells were stained with CD3 V450 (blue). Nuclei were left unstained. To confirm appropriate staining of the necrosis and hypoxic markers during optimization, confocal microscopy imaging was also performed on BAL cells (g) under basal culture conditions (untreated) and (h) under experimentally induced hypoxic conditions (treated) using traditional 2D culture methods, and BAL cells were stained with HMGB1 AF647 (red), HIF-1α AF488 (green), and Hoechst (nuclei). Single stains of (i) the HIF-1α and (j) the HMGB1 markers under chemically induced hypoxic conditions demonstrated unsuccessful capturing of HIF-1α nuclear translocation during hypoxia but successful capturing of cytoplasmic translocation of HMGB1 proteins, indicative of necrosis. Confocal microscopy imaging of (k) the entire 3D human TB granuloma section stained with HMGB1 (red) demonstrated the establishment of an oxygen gradient resulting in (l) an AM core of both necrotic and nonnecrotic cells and (m) a cuff of nonnecrotic cells.

Confocal microscopy (Fig. 2a) demonstrated that both the outer edges (Fig. 2b) and core (Fig. 2c) of the innate granuloma are made up of predominantly AM (CD206 PE-CF594 [red]). A tile scan (Fig. 2d) of the adaptive granuloma midsection revealed a cuff of autologous CD3^+^ T cells (Fig. 2e) and an AM-dominant core (Fig. 2f), again demonstrating little-to-no autologous CD3^+^ T cells infiltrating the core. While the granuloma core composition varies depending on the stage of the granuloma development, our findings can be considered synonymous with the inferences made about human granuloma structures from nonhuman primate (NHP) models of TB, at least at certain time points during their development (19, 20). Considering recent data suggesting that AM serve only as an early niche to productive M. tuberculosis infection, whereafter recruited monocyte-derived macrophages and neutrophils become the main infected cell type, it is likely that the AM core of the spheroid granuloma represents an early granuloma state (1, 21), guiding the support for the use of this model above NHP models.

In addition to our 3D spheroid granuloma structures being morphologically relevant, it remains important to use this model to recapitulate physiologically relevant events of *in vivo* granulomas. It is well known that TB granulomas undergo structural and localized changes during development and maturation. These changes often include the caseation of the core through the process of necrosis, which contributes to morbidity by causing tissue damage. The caseous granuloma *in vivo* is notably hypoxic, driving the accumulation of hypoxia inducible factor 1-α (HIF-1α), and considered the hallmark of failed M. tuberculosis containment, implicated in transmission (22–24). HIF-1α, which is ubiquitously expressed in the cytoplasm under normoxic conditions, is used in this study as a hypoxia target (25), while necrotic cells are known to release the high-mobility group box 1 (HMGB1) protein from the nucleus into the cell cytoplasm (26). We compared the expression of HMGB1 and HIF-1α in our 3D spheroid granuloma model to that in traditional control cultures. Traditional cell culture controls created using BAL cells in an AnaeroPack experiment displayed basal expression patterns for both targets, synonymous with cells being nonnecrotic and normoxic (Fig. 2g). Following experimental manipulation of traditional cultures to induce a hypoxic environment (Fig. 2h), an upregulation in cytoplasmic expression of HIF-1α, although no nuclear translocation (indicative of a hypoxic cell), was observed (Fig. 2i); however, the cytoplasmic translocation (indicative of a necrotic cell) of HMGB1 from the nucleus to the cytoplasm of BAL cells was achieved in traditional culture after experimental induction (Fig. 2j). In contrast, we investigated both targets within our 3D spheroid granuloma model (Fig. 2k), which did not require any additional experimental manipulation whatsoever, such as was necessary for the traditional cell culture controls. Here, we demonstrated that cytoplasmic translocation of HMGB1 successfully occurs in BAL macrophages when they are located at the core of the 3D spheroid granuloma (Fig. 2l) and remains in the nucleus of T cells at the cuff (Fig. 2m), displaying a brightly stained rim (27). We were unsuccessful in capturing any HIF-1α immunofluorescent signals within the 3D spheroid granuloma model, and we propose that this is as a result of HIF-1α being highly ubiquitinated upon reexposure to oxygen, making it difficult to target without knowing the point at which hypoxia is induced during M. tuberculosis infection (28). Our data confirm that the core of the 3D spheroid granuloma contains AM, which are both necrotic and nonnecrotic, and that a cuff of nonnecrotic T cells surrounds this core, recapitulating both morphological and physiological characteristics of TB granulomas. While traditional cell cultures require experimental manipulations for the induction of hypoxic and necrotic responses seen *in vivo*, our 3D human spheroid granuloma can recapitulate the *in vivo* granuloma microenvironment without external manipulation by mimicking the spatial organization and cellular interactions within the 3D conformation, which may allow for the appropriate investigation of these forms of cell death.

### Single-cell immune phenotyping of postculture granuloma cells reveals maintained viability and distinct immune phenotypes.

**FIG 3:**
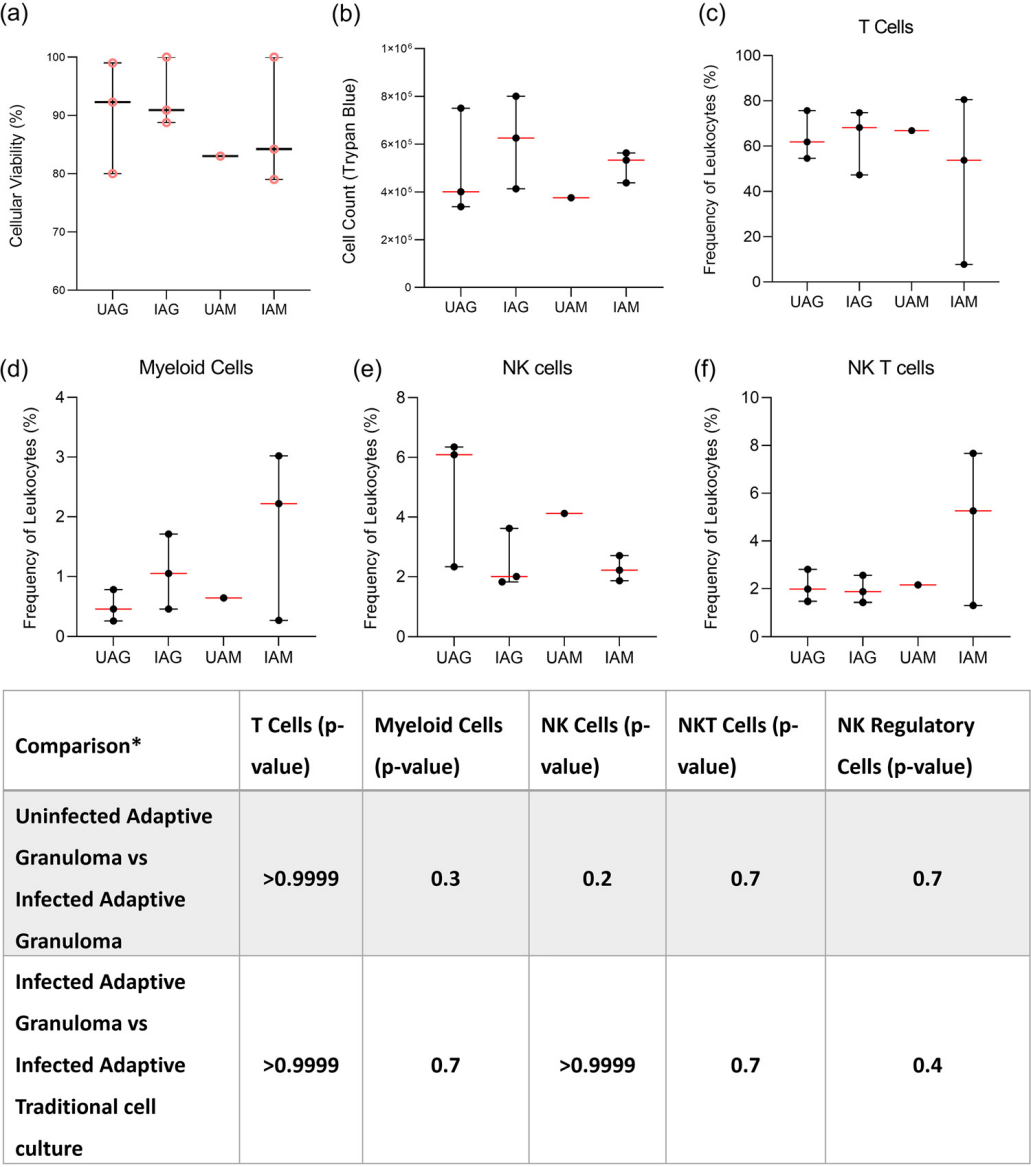
From each granuloma structure from each participant, the viability of mechanically dissociated granuloma structure single cells could be determined using the (a) trypan blue exclusion method (pink, circular data points). (b) Viable cells could be counted using the trypan blue exclusion method, and using flow cytometry, the frequency of various immune cell phenotypes could be assessed after the 48-h adherence period to remove AM, including (c) CD3^+^ T cells, (d) CD3^−^CD14^+^ myeloid cells, (e) CD3-CD16^+^ natural killer (NK) cells, and (f) CD3^+^ CD16^+^ NK T cells. Cell counts and viability data are representative of cellular integrity after the final 48-h incubation for adherence for both uninfected (*n* = 3) and infected (*n* = 3) granuloma structures, as well as uninfected (*n* = 1) and infected (*n* = 3) traditional cell cultures (monolayer). Each point represents a single data point; error bars are representative of the median and range. UAG, uninfected adaptive granuloma; IAG, infected adaptive granuloma; UAM, uninfected adaptive monolayer; IAM, infected adaptive traditional. Mann-Whitney *t* test results for comparisons between infection groups for each cellular phenotype are given in the table below the graphs. *, comparisons could not be made between the uninfected adaptive granuloma and uninfected adaptive monolayer groups and the uninfected adaptive monolayer and infected adaptive monolayer groups due to too few data points being available for the uninfected adaptive monolayer group.

The viability and cell number of mechanically disrupted 3D granuloma structures were assessed via flow cytometry and compared to those of traditional cell culture controls. Innate granuloma structures could not be assessed in this manner owing to the highly autofluorescent nature of AM, making flow cytometry near impossible on these cells (Fig. S1). Particulate matter found within the AM of smokers or persons exposed to biomass fuels appears to be responsible for the autofluorescence, as is demonstrated by signals observed in the red channel during lambda scanning on the confocal microscope (Fig. S2) (29). Importantly, we could demonstrate that it was the AM and not the CD3^+^ T cells which were autofluorescent. Therefore, only adaptive granuloma structures were mechanically dissociated after the full culture period of 6 days, as these could then be cultured for adherence to separate the AM from the T cells. Following prolonged culture, we have shown that the AM core becomes necrotic, and thus we expect the cells to dye more easily with trypan blue compared to traditional cell culture controls. Using the trypan blue exclusion method, cell viability (Fig. 3a) and cell number (Fig. 3b) could be assessed and compared for each structure. No statistically significant differences were observed between the four groups for either cell viability or cell counts based on the small sample size.

10.1128/mSphere.00552-21.1 FIG S1 (a) Unstained total BALC run on the BD FACS Canto II demonstrate positive autofluorescent signals in multiple channels, with the final scatter plot demonstrating an overlay of the unstained BALC and BALC stained with CD206 AF647, compared to (b) unstained PBMC which demonstrate no autofluorescence. (c) Removing nonadherent cells from the adherent macrophages results in the ability to stain other lymphoid cells like CD3 without autofluorescent interference. Gates were set using FMO quality control checks set using a PBMC sample. Download FIG S1, PDF file, 0.2 MB. Copyright © 2021 Kotze et al. 2021 Kotze et al. https://creativecommons.org/licenses/by/4.0/ This content is distributed under the terms of the Creative Commons Attribution 4.0 International license.

10.1128/mSphere.00552-21.2 FIG S2 (a) Preliminary staining of alveolar macrophages with Hoechst demonstrated autofluorescent signals in the red, green, and blue channels, (b) with the autofluorescent signal in the red channel originating from the particulate matter-containing vesicles identifiable under brightfield and the overlay (as indicated by arrows). (c) Cytospin of CD3^+^ T cells stained with CD3 V450 only showed surface staining of the CD3 receptor. (d) Unstained granulomas show distinct autofluorescent signals in the red and blue channels; however, (e) when granulomas are stained with CD3 V450 alone, distinctly stained rings are observed for the T cells, as confirmed using transmitted light, which also confirms the lack of particulate matter within the T cells. Download FIG S2, PDF file, 0.1 MB. Copyright © 2021 Kotze et al. 2021 Kotze et al. https://creativecommons.org/licenses/by/4.0/ This content is distributed under the terms of the Creative Commons Attribution 4.0 International license.

We then investigated the ability to conduct single-cell immune phenotyping on deconstructed granuloma single cells following 6 days of culture. We also wanted to know how the 3D conformation and resultant molecular signals would affect cell frequencies compared to traditional cell culture cultures. From the same mechanically dissociated adaptive granuloma structures and corresponding traditional cell culture controls as used for the assessment of viability, phenotyping by way of flow cytometry (FACS Canto II) could be successfully performed on the nonadherent cell fraction. Autologous CD3^+^ T cells (Fig. 3c), CD3^−^ CD14^+^ myeloid cells (Fig. 3d), CD3^−^ CD16^+^ NK cells (Fig. 3e), and CD3^+^ CD16^+^ NKT cells (Fig. 3f) could be identified from the nonadherent cell fraction, with CD3^+^ T cells proving to be the dominant cell subset. While none proved statistically significant (table in Fig. 3), there were visible differences in the phenotypic profiles of the nonadherent cell fractions of the uninfected and infected adaptive granuloma structures, as well as the uninfected and infected traditional cell culture controls (referred to as a “monolayer” within the figure). While the proof-of-concept nature of this study does not allow for strong biological inferences made based on the results observed for the granuloma structures and traditional cell cultures, our study does show promise for future investigations where we plan to significantly increase the number of granuloma structures assessed.

### 3D granulomas secrete proinflammatory cytokines into the extracellular environment, signaling the recruitment of other immune cells.

**FIG 4:**
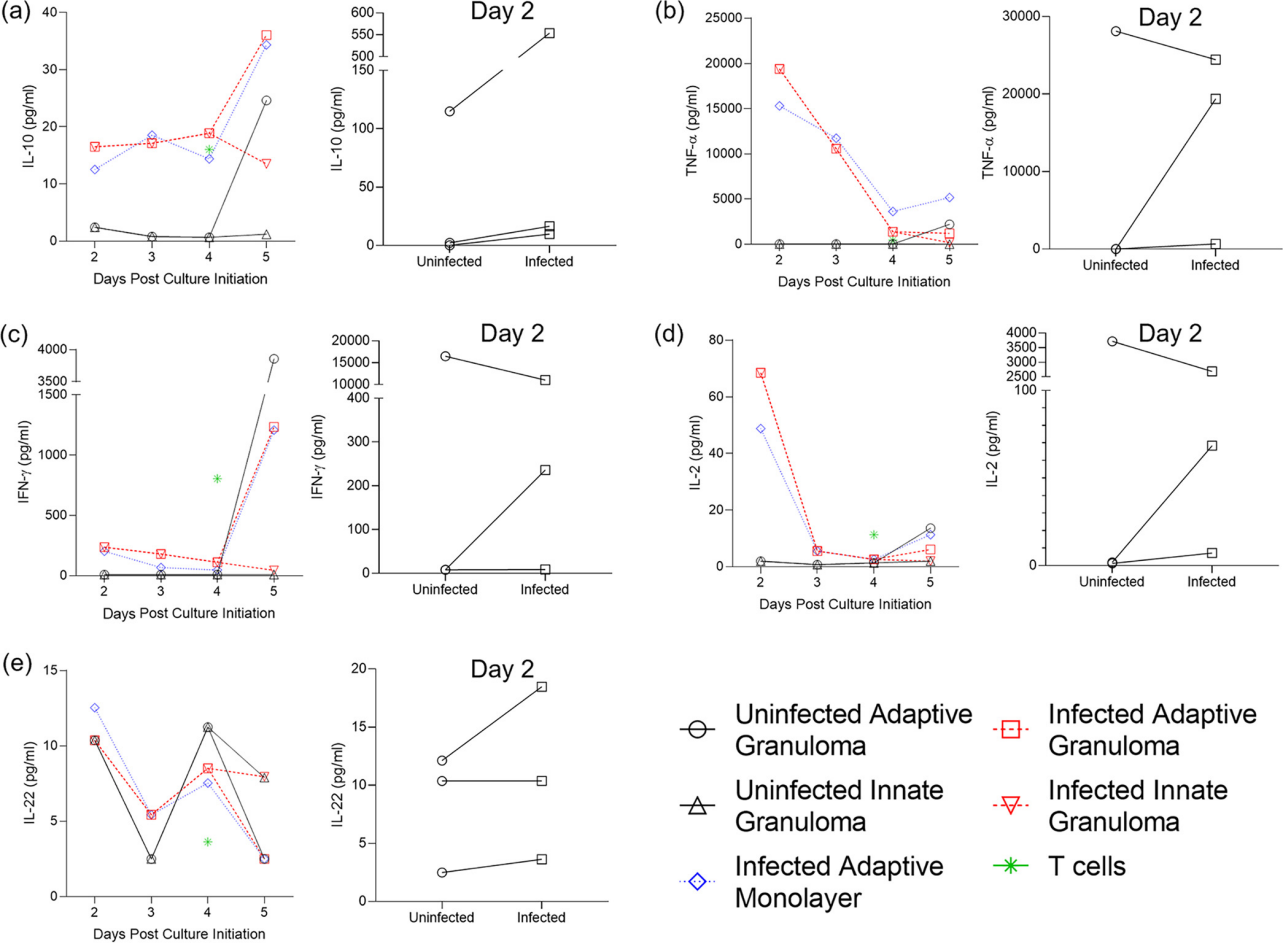
Concentration (pg/ml) of the investigated cytokines released into the supernatant of the 3D granuloma structure and traditional cell culture control (monolayer) extracellular environments, as measured by Luminex analysis. The cytokines measured included (a) IL-10, (b) TNF-α, (c) IFN-γ, (d) IL-2, and (e) IL-22. Cytokine production was compared between initial production by uninfected and that by BCG-infected AM 2 days post culture initiation and subsequent release until the end of culture (5 days post culture initiation), as well as compared to the corresponding cytokine release by the BCG-infected chronic traditional cell culture control (monolayer) and autologous CD3^+^ T cells prior to addition to the AM culture. Each data point represents the median of three individual participants, with BCG infection occurring on day 1 after culture initiation (the day of culture initiation is considered day 0). The day 2 inset for each cytokine depicts the differences between cytokines released by uninfected and BCG-infected AM 2 days post culture initiation, i.e., 1 day postinfection (each data point represents a single individual from the three individual participants assessed).

Traditional cell cultures are known for their capabilities of producing cytokines and chemokines during short-term cultures, as measured frequently by Luminex immunoassays. Supernatants from 3D granuloma structures and traditional cell culture controls were harvested throughout the culture period and at the end of the 6-day culture. The most interesting differences to investigate would be the production of the selected cytokines from both AM and autologous CD3^+^ T cells, comparing these between 3D granuloma structures and traditional cell culture controls. We performed a Luminex immunoassay on the harvested supernatants and measure the concentrations of interleukin 10 (IL-10) (Fig. 4a), tumor necrosis factor alpha (TNF-α) (Fig. 4b), gamma interferon (IFN-γ) (Fig. 4c), IL-2 (Fig. 4d), and IL-22 (Fig. 4e) (raw data can be found in Table S1). Tests for normality could not be performed due to the small sample size, but QQ plots of the data showed that the data could follow a Gaussian distribution if more data points were available. Data were, therefore, treated as nonparametric data, with the Kruskal-Wallis test being performed using Dunn’s posttest to correct for multiple comparisons. All cytokines assessed could be successfully measured from the harvested supernatants, with the cytokine IFN-γ showing high production in T cells prior to their addition to the granuloma structure, as would be expected from a normal culture. While none of the cytokines showed statistically significant differences between the groups, this was expected due to the low sample size but was performed regardless to demonstrate the viability of the generated 3D granuloma structures over a long culture period and that they interact in a manner reminiscent of the *in vivo* human TB granuloma. Of particular interest was the observation that the innate granulomas (both uninfected [UIG] and BCG-infected [IIG]) did not produce high levels of cytokines like TNF-α, IFN-γ, and IL-2 associated with T cell production and release, further supporting the previous statement. While not significantly different, the differences between the 3D granuloma structures and the traditional cell controls could prove to be higher in the 3D granuloma structures should we increase the sample size, as a few differences were already apparent, for example IFN-γ.

10.1128/mSphere.00552-21.5 TABLE S1 Raw Luminex data for the participants TBH7760, TBH6999, and TBH1252, plotted in Fig. 4. Download Table S1, PDF file, 0.1 MB. Copyright © 2021 Kotze et al. 2021 Kotze et al. https://creativecommons.org/licenses/by/4.0/ This content is distributed under the terms of the Creative Commons Attribution 4.0 International license.

### 3D adaptive granuloma structures regulate mycobacterial replication.

**FIG 5:**
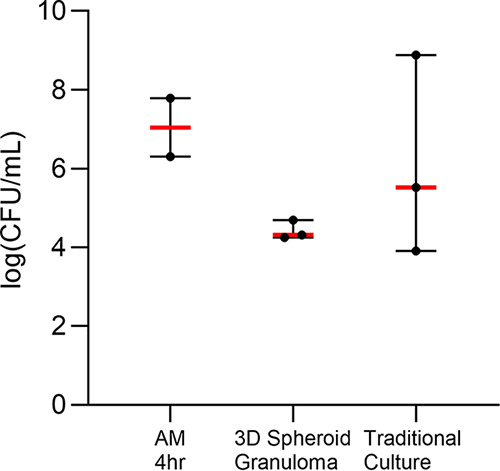
BCG CFU were measured using the cell lysate of 3D spheroid granuloma structures and traditional cell culture control cultures (traditional cultures) and compared to the initial bacterial uptake of BCG into AM. Data were log-transformed prior to plotting.

When total cell numbers from patient samples were not limiting, CFU were measured for each granuloma structure using the cell lysate (Fig. 5). For the purposes of this pilot study, we were able to measure the initial uptake by AM after the 4-h infection from two of the participants, which allowed for an indication of bacterial uptake prior to the long-term culture of the 3D granuloma structures (*P* = 0.2) and traditional cell culture controls (*P* = 0.8). Due to a lack of adequate cell numbers, the BCG-infected innate traditional cell culture control had a single data point only and was therefore excluded from analysis. The log(CFU/ml) of the BCG-infected adaptive 3D granuloma was lower than that of the BCG-infected traditional cell culture control, but this was not significant (*P* = 0.7). With added future experiments, this model has the potential to elucidate differences in bacterial control and containment at different stages of granuloma development, and we are sure that the 3D spheroid granulomas will prove to be more protective than traditional cultures, more accurately representing the *in vivo* granuloma environment and supporting the use of a 3D spheroid granuloma over traditional culture.

### Mycobacterial infection and spheroid configuration both alter the gene expression of cells in 3D spheroid granulomas.

**FIG 6:**
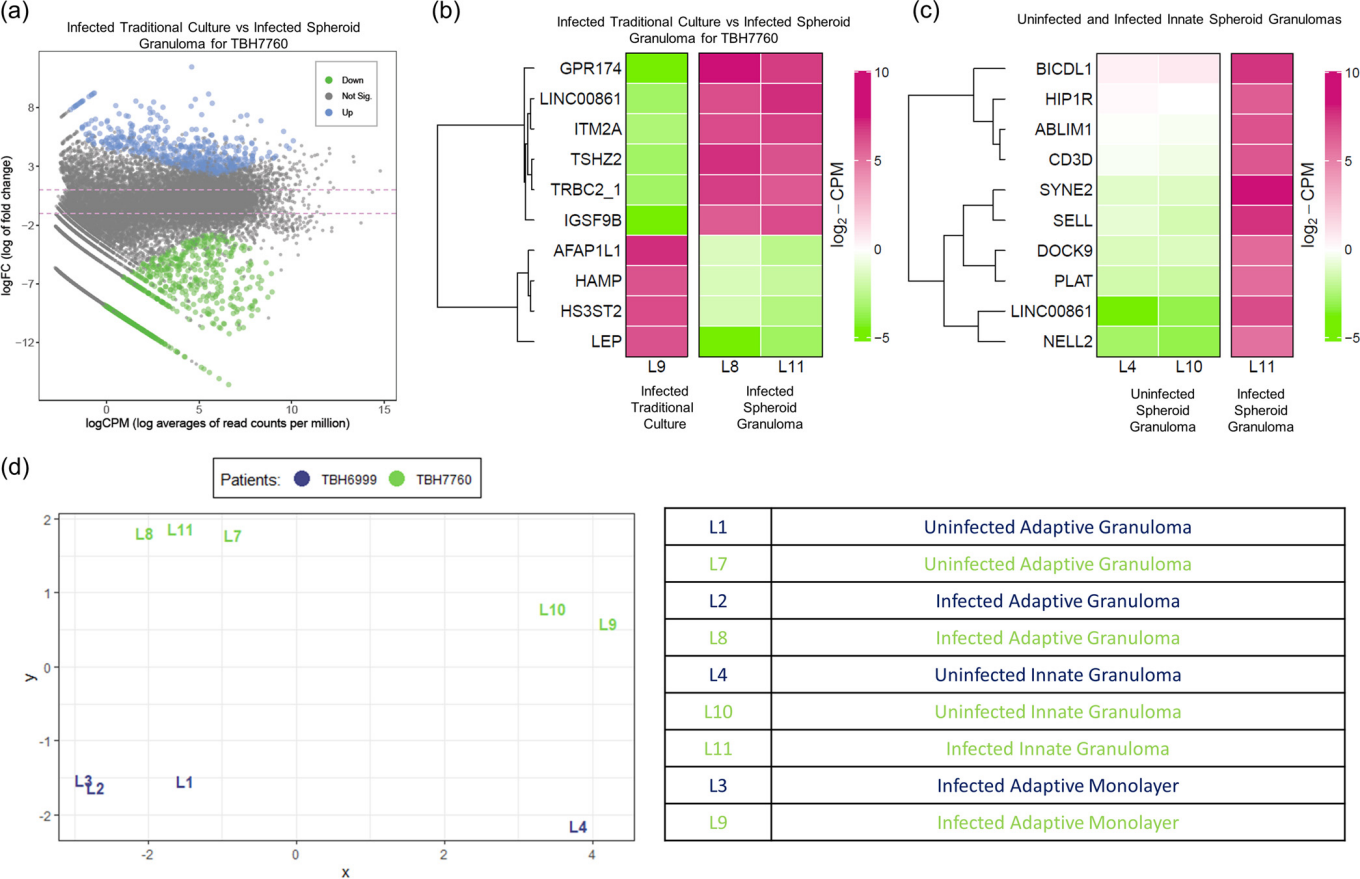
RNA sequencing relative gene expression results displaying (a) a mean-difference (MD) plot of the differential gene expression between BCG-infected traditional control cultures and 3D spheroid granulomas (the distance between any two points is the leading log-fold change between those samples; the leading log_2_ fold change is the root mean square average of the largest log_2_ fold change between those samples), (b) a heatmap of the differential gene expression of BCG-infected cells in traditional culture (labeled “Control”) versus that of corresponding infected cells in 3D spheroid granulomas, (c) a heatmap of the differential gene expression in BCG-infected innate 3D spheroid granulomas versus that of uninfected innate 3D spheroid granulomas, and (d) a multidimensional scaling plot of all data points from a smoker (green) versus those from a nonsmoker (blue). Descriptions of each data point are given in the bottom right-hand table.

We evaluated the ability to extract quality RNA from individually dissociated 3D spheroid granulomas and conducted bulk RNA sequencing on the cells. Total RNA was extracted from 11 available samples, but 2 did not meet the total RNA requirement for acquisition on the MGISEQ-2000; therefore, only 9 samples were used for further evaluations (table in Fig. 6). FASTQC was used to assess the quality of the 18 FASTQ files generated, and manual inspection of the quality score graphs of all 18 HTML reports showed that the lowest 10th percentile value for any base at any position was 26; in all cases, the software issued a passing grade (Fig. S3a, b, d, and e). Another important quality metric to consider is the proportion of bases seen at each position. All 18 reads (9 forward and 9 reverse reads) showed erratic behavior in the first 10 to 13 bases before a transition to a smooth curve for the rest of the read (Fig. S3c and f). For transcriptome sequencing (RNA-Seq) data, this is expected and is a result dependent on the specific library kit which was used. As a result, further cleaning or processing of the raw FASTQ files (for example, trimming of adaptor sequences) was not required.

10.1128/mSphere.00552-21.3 FIG S3 Representative FASTQC quality control results of the nine forward (a to c) and nine reverse (d to f) reads for the nine patient samples run on the MGISEQ-2000. Representative results include (a) the average quality score per base for all nine forward reads, (b) forward read quality score per sequence, (c) an example of the proportion of bases seen at each position for the forward reads where the first 10 to 13 bases show erratic behavior, (d) the average quality score per base for all nine reverse reads, (e) reverse read quality score per sequence, and (f) an example of the proportion of bases seen at each position for the reverse reads where the first 10 to 13 bases show erratic behavior. Download FIG S3, PDF file, 0.2 MB. Copyright © 2021 Kotze et al. 2021 Kotze et al. https://creativecommons.org/licenses/by/4.0/ This content is distributed under the terms of the Creative Commons Attribution 4.0 International license.

The geometrical impact of 3D granulomas on the biological responses of immune cells remains unexplored. We therefore compared the gene expression profile of the 3D spheroid granuloma to that of the corresponding human subject’s cells (matched cell origin, types, ratios, numbers) in traditional cell culture. Results from this demonstrate that cells in 3D spheroid granulomas upregulate 640 genes and downregulate 523 genes compared to those in traditional culture (Fig. 6a). Loose inferences based on the top 10 differentially expressed genes (Fig. 6b) suggest that 3D spheroid granulomas could augment the transcription of proteins related to inhibitory synapse development, a neuropeptide receptor, a zinc finger transcription factor, and T cell activation but curb those related to leptin, hepcidin, actin filament proteins, and a heparan sulfate-glucosamine enzyme. In total, we identified 138 genes with significant differential expression between BCG-infected 3D spheroid granulomas and BCG-infected traditional culture. These 138 differentially regulated transcripts were functionally annotated to gain an overview of the biological pathway regulation using GO enrichment analysis. The REVIGO resource was used to summarize and visualize the most enriched GO terms for biological processes (Fig. S4a), molecular function (Fig. S4b), and cellular components (Fig. S4c) in an interactive graph. Differential gene expression was also observed between uninfected and BCG-infected 3D spheroid granulomas when innate 3D spheroid granulomas were investigated (Fig. 6c), with the top 10 upregulated genes representing pathways linking organelles to the actin cytoskeleton for subcellular spatial organization, a cytoskeletal protein binding actin, proteins of the secretory vesicle machinery and intracellular signaling, components of T cell engagement, tissue-type plasminogen activator, l-selectin, neural epidermal growth factor, and huntingtin-interacting protein. A multidimensional scaling plot of all data points demonstrated differences between both participants from which RNA-Seq samples were available as observed by the grouping in the first component between the two different participants (Fig. 6d, smoker [green] versus nonsmoker [blue]). Differences in the BCG-infected 3D spheroid granulomas between participants were not significant, with no genes achieving a false-discovery rate of less than 0.05, as with the differences observed above.

10.1128/mSphere.00552-21.4 FIG S4 Functional network map of the GO term enrichment analysis for the 138 differentially regulated proteins identified during RNA-seq analysis from comparing BCG-infected spheroid granulomas between participants (one a smoker and one not). GO terms retrieved by the Database for Annotation, Visualization, and Integrated Discovery (DAVID) and visualized in REVIGO clustered enriched terms according to (a) biological processes, (b) molecular function, and (c) cellular component. Each node corresponds to a single representative GO term for all related sibling and child terms. Highly similar GO terms are linked by edges in the graph where line width indicates the degree of similarity. Bubble color intensity indicates the *P* value and bubble size indicates the frequency of the GO term in the GOA database. Force-directed layout algorithm was used to keep similar nodes together. Download FIG S4, PDF file, 0.07 MB. Copyright © 2021 Kotze et al. 2021 Kotze et al. https://creativecommons.org/licenses/by/4.0/ This content is distributed under the terms of the Creative Commons Attribution 4.0 International license.

While these results provide the groundwork for our model’s inception, our RNA-Seq data demonstrate the ability to successfully isolate and sequence high-quality RNA from the 3D spheroid structures after prolonged culture and infection. The preliminary data allude to differential behavior of host immune cells based on structural organization and conformation upon BCG infection, as observed in the differences between traditional cell culture and the 3D spheroid structures, which may, in future, lead to the possibility of demonstrating that traditional cell culture methods do not accurately reflect responses occurring at a granuloma level during mycobacterial infection. Since this study developed the method of generating 3D spheroid granulomas, future studies will include the comparison of larger numbers of participants to investigate this hypothesis.

## DISCUSSION

Human responses to M. tuberculosis infection range in complexity, while the heterogeneity of TB disease is unprecedented and challenging to model. Most infected individuals can mount a protective immune response to control infection, resulting in a large proportion of these individuals achieving sterilizing cure. Yet, a small proportion will develop active TB disease, and some will retain a latent infection where they are able to control infection but not achieve sterilizing cure (7, 30, 31). Even within individuals, granulomatous lesions present as dynamic and localized microenvironments within the lung, each with their own unique organization and ability to control infection, ranging from sterilizing cure to immune failure (5, 7, 32, 33).

A major knowledge gap is the exact nature, function, and spatial organization of immune cells constituting protective granulomas. A hallmark of progression to active TB disease is changes to granuloma physiology, such as an increase in granuloma number and distribution, as well as changes in granuloma function, such as poor M. tuberculosis replication control and development of central necrosis and cavitation. These signify the host’s inability to eliminate bacilli and are indicative of failed immunity. As an added complexity, differences exist in the rate and trajectory of granuloma progression, which is determined by features such as host immunity and bacterial virulence (34–36). Therefore, TB patients simultaneously harbor a range of granulomas, comprising a spectrum of solid nonnecrotizing, necrotic, and caseous granulomas, each with its distinct kinetics. Although the overall clinical picture is likely defined by a set of the most poorly performing granulomas in a particular host, granulomas from a single individual vary considerably in cellular composition, cytokine profile, morphology, immune phenotype, and bacterial burden. It is therefore important to describe host responses at the individual granuloma level.

While animal models have contributed significantly to our understanding of the mammalian immune system, numerous examples have shown that laboratory species do not faithfully or in full mimic human immunity or TB disease (10–12). There are murine models available which develop necrotic lesions when infected with M. tuberculosis; however, not all murine species are in this way appropriate (27, 37, 38). A major shortfall of murine TB granuloma models is the considerable immunologic disconnect between murine and human macrophage biology and the limited ability to control bacterial replication, with the largest disconnect stemming from their differences in inducible nitric oxide synthase (iNOS) expression and nitric oxide (NO) production, making these models unsuitable for human inference (39–41). Other species models such as rabbits and nonhuman primates have been useful in studying lung pathologies, but these models are expensive and laborious to maintain, and biological reagents are limited. The heterogeneity in host responses to M. tuberculosis infection is currently more readily investigated in animal models such as cynomolgus macaques, with findings that are translatable to human TB. As such, our understanding of the structure and function of human TB granulomas is largely hinged on features reported for NHP TB granulomas.

**FIG 7:**
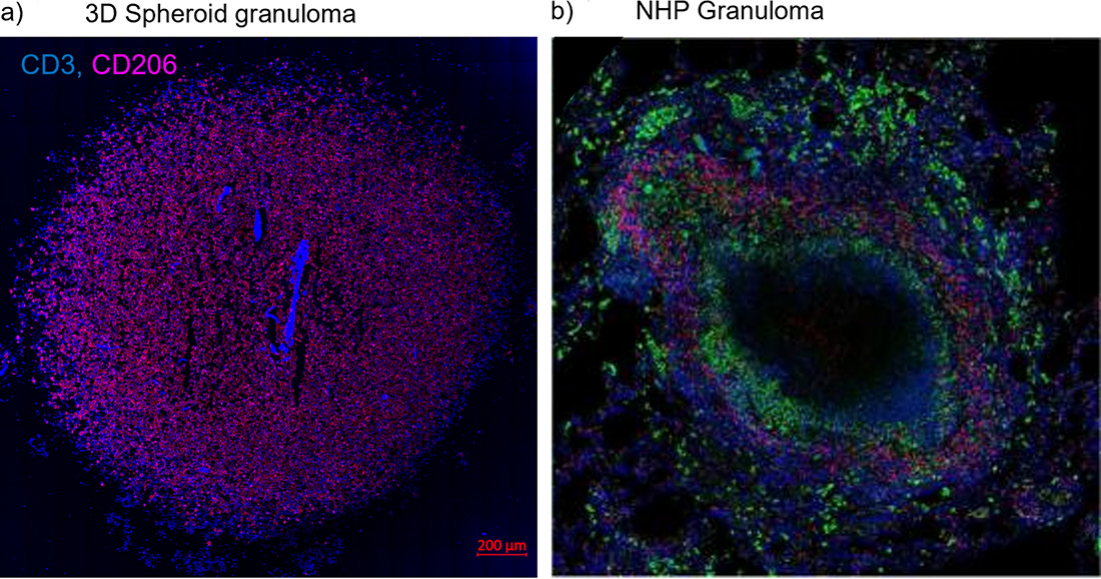
Our 3D *in vitro* TB granuloma structures show similar structural and cellular composition based on (a) immunofluorescence staining with antibodies for CD3^+^ T cells (blue, V450) and alveolar macrophages (red, PE-CF594) to (b) published *in vivo* TB lung granulomas of nonhuman primates, stained with antibodies for CD3^+^ T cells (red) and CD68^+^ macrophages (green), surrounding the necrotic center (unstained). Adapted from Flynn et al. 2015 with permission (license number: 4967651365284) (19).

Our 3D adaptive spheroid granuloma has here been demonstrated to be similar in structure and features to NHP TB granulomas, as is evidenced by the spatial arrangement of an autologous T-cell cuff surrounding a predominantly macrophage core (Fig. 7) (19, 20, 42). While our model specifically stained for CD206^+^ AM, it is well understood that the stages in granuloma progression are characterized by an ever-changing macrophage milieu; as such, our model can be considered a reflection of the early stages of granuloma progression, and future experiments using this model should investigate the progression of the granuloma further, specifically investigating the proportions of tissue-resident macrophages (CD68^+^) and other subsets like monocyte-derived macrophages to the here-reported alveolar macrophage subset (1, 43). This model allows for manipulation of biological targets and, importantly, captures *in vivo* characteristics, such as spatial organization as observed in *in vivo* human and NHP granulomas. This could have multiple benefits over traditional culture methods and enable assessment of host responses to M. tuberculosis in the context of intricate cellular interactions and visualization of granuloma organization through 3D and quantitative analysis. The development of granuloma models that accurately reflect the major pathophysiological conditions existing in the spectrum of *in vivo* human pulmonary TB granulomas, both kinetically and in different clinical phases of infection, is imperative. Ideally, this would require the use of cells derived from patients within the spectrum of TB disease and be retrieved from the site of disease to fully recapitulate physiological events occurring within the human lung when challenged with M. tuberculosis. We have established a novel, laboratory-based, biologically relevant platform for generating patient-derived 3D spheroid granulomas mimicking human TB. The platform enables analysis of genomic, epigenetic, immunologic, structural, and pathogen- and treatment-specific aspects of immune cells during granuloma evolution to resemble human pulmonary TB lesions more closely. The 3D spheroid granuloma model is assembled as a single, organized structure in a culture well, consisting of human lung-derived AM, surrounded by layers of autologous, peripherally recruited T cells. This model has the potential to replicate characteristics observed during granuloma evolution (17, 44), such as hypoxia and nutrient concentration gradients, and for in-depth mechanistic analysis of crucial lung granulomatous features observed in the spectrum from latent to active TB.

Granulomas are known to be dynamic, organized structures with gene expression and epigenetic profiles correlating with lesion type and developmental trajectory. Considering the limitations associated with *in vivo* human research, few studies have explored the immune environment within human lung granulomas (45–47). Results have, however, demonstrated that pooled analysis of different human lung granuloma types may underestimate differences in gene expression specific to each lesion, revealing a higher number of significantly differentially expressed genes in fibrotic nodules than in the cavitary granulomas (48). Thus, a distinct gene expression pattern was observed for each granuloma type or stage, and increased methylation was found in granulomas of patients with more severe disease (49–51). Our granuloma model has alluded to these observations, with the extraction of good quality RNA from this model proving that structures generated from individuals along the spectrum of disease are likely capable of providing detailed gene expression profiles for the various stages of infection.

The first guiding 3D model described for M. tuberculosis infection was described by Puissegur et al. in 2004 and has helped direct current model strategies (52). Since then, a number of models have been established with varied success rates (reviewed by Elkington et al., 2019) and with little focus being placed on primary human cells obtained from the site of disease, one of the proposed requirements of an optimal model (9, 14). Most popularly, *in vitro* granuloma-like cell aggregates established using M. tuberculosis-infected peripheral blood mononuclear cells (PBMC) have shown promise in generating cellular aggregates which mimic granuloma formation (53, 54). While easy to establish and high-throughput, this model of infected PBMC forms multiple structures within a culture well, each structure at a different “stage” of granuloma development, which may limit some aspects of granuloma investigations (36). PBMC granuloma models also do not accurately represent the immune cell milieu and composition, or the defined organizational structure, as observed for the 3D spheroid model. In addition to this, we have demonstrated with various molecular tools the validity of employing such a model for the investigation of M. tuberculosis infection without the limitations of traditional cell culture methods and the need to acquire entire *in vivo* structures from procedures such as biopsies. The most recently published *in vitro* granuloma model example using biopsy samples includes an adult stem cell-derived airway organoid developed from cells retrieved from human lung biopsies (55). The limitation of such a model is that it requires difficult-to-obtain sample types and complex organoid generation methods which do not include the use of primary phagocytes such as alveolar macrophages, which are essential for the initial control of M. tuberculosis infection. Another important consideration for our model is that granuloma formation is not limited to M. tuberculosis infection but also occurs in several chronic infections, including *Schistosoma* spp., Salmonella enterica, and Listeria monocytogenes, and noninfectious diseases like sarcoidosis (53), which together cause millions of deaths. Our granuloma model could therefore be adapted to depict these disease-specific granulomatous features. Finally, an important strength of this model is the ability to assess the earliest granuloma states, interhost variability in alveolar macrophage responses, and variability in macrophages with different vaccination states, coinfections, and comorbidities.

Our model is not without its limitations, however. The autofluorescent nature of alveolar macrophages from the lungs of individuals in certain regions like Cape Town, South Africa displays high carbon particulate matter which results in autofluorescence. We have demonstrated that this limitation can be circumvented; alternatively, molecular techniques like CyTOF (cytometry by time of flight) could be used to investigate both the AM and autologous CD3^+^ T cell fractions together, without the need to consider autofluorescence, as has previously been demonstrated by our group (29). The use of the magnetic levitation drive, for one, prevents the unrestricted movement of NanoShuttle-labeled AM until the core is stable, and therefore features of early AM movement could be missed. This model was established using BCG as a model for TB owing to the benefits of being able to work with this organism outside a BSL3 environment. Considering the physiological differences between BCG and H37Rv, the virulent laboratory strain of M. tuberculosis, this model needs to be validated in a setting whereby H37Rv is used as the infectious agent instead of BCG. We are confident, however, that the model we have established using BCG as a model organism for M. tuberculosis will be translatable for not only M. tuberculosis but other pulmonary pathogens which result in the formation of pulmonary granulomas. As such, the results generated in this study during the establishment of the 3D spheroid *in vitro* granuloma model should function to inform the scientific community of the possibilities for which this model can be adapted.

Finally, our 3D spheroid *in vitro* granuloma model still requires comparative assessments to *in vivo* granulomas with respect to the cellular components, cell phenotype, molecular and epigenetic interactions, patterns of cytokine and chemokine secretion, mycobacterial dormancy, subcellular dissemination, and ultimately impact on clinical outcome. These are currently ongoing through planned human and nonhuman primate studies, along with a study whereby we have begun adding additional cell types, such as B cells, monocytes, and myeloid-derived suppressor cells (MDSCs), to the 3D spheroid granuloma as one would expect to see in a developing granuloma (6, 56, 57). In parallel, we are also conducting a study whereby we investigate the differences in granulomas created using AM versus monocyte-derived macrophages to investigate differences in early- and late-stage granulomas. With that said, the current model would provide insights into host-mycobacterial interactions at stages too early to address within such *in vivo* models and, eventually, serve as a preferred platform for initial preclinical testing of TB vaccine and drug candidates.

## MATERIALS AND METHODS

### Study subjects.

This proof-of-concept study enrolled three HIV-uninfected participants between the ages of 18 and 70 years who presented to the Pulmonology Division of Tygerberg Academic Hospital with clinical indications of TB. These largely included clinical and radiological abnormalities as determined by chest X-ray (CXR). Mycobacteria growth indicator tube culture (MGIT) and/or GeneXpert MTB/RIF was performed. Written informed consent was obtained from all participants, and a summary of their demographics is given in Table 1. The study design was approved by the Stellenbosch University Ethics Review Committee (IRB number N16/04/050).

**TABLE 1:** Demographic details of the participants used for the generation of *in vitro* 3D granulomas^*a*^

Characteristic	Detail for participant ID		
	TBH7760	TBH6999	TBH1252
Age^*b*^	43	34	51
HIV status	Negative	Negative	Negative
Sex	Female	Male	Male
Smoking habits	Smoker	Nonsmoker^*c*^	Smoker
Pellet color^*d*^	Black	Black	Black
Diagnosis	Interstitial fibrosis (smoking-related)	Sarcoidosis	Interstitial lung disease
TB history	Previous TB	Previous TB	No previous TB
BALC count	6.67E07	2.07E06	4.18E06
Existing treatment	Nifedipine	Amoxicillin, co-amoxiclav	None
Existing conditions	Raynaud’s phenomenon	Unknown	Hypogonadism

a Abbreviations: ID, identification; BALC, bronchoalveolar lavage cell.

b Age at time of enrollment.

c Stopped smoking within the last 3 months.

d Pellet color has been shown to be representative of participant smoking habits and exposure to biomass fuels.

**FIG 8:**
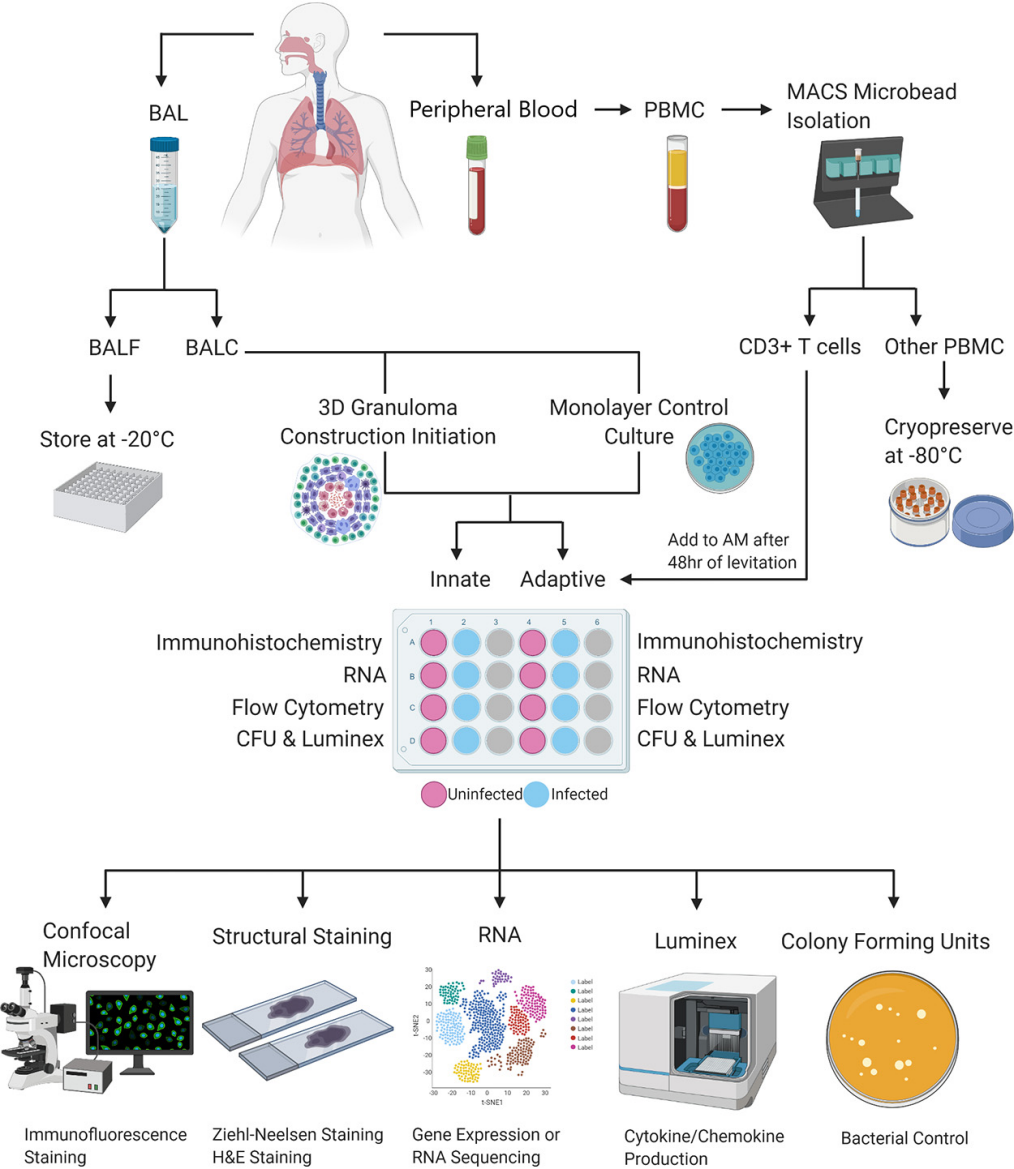
A basic representation of the workflow used to generate and analyze 3D *in vitro* human TB granulomas generated from a single participant. Briefly, BAL fluid and peripheral blood are collected from the participant at the time of the bronchoscopy procedure, after which the BAL fluid is processed to collect the cell pellet while the fluid is stored for other studies. The BAL cells (BALC) are then used to construct the alveolar macrophage core of the 3D structure and the traditional cell culture control (monolayer) in both uninfected and infected scenarios. Collected peripheral blood is processed for PBMC and then further processed to isolate autologous CD3^+^ T cells using the MACS MicroBead cell separation technique, with the CD3^−^ cellular fraction being stored for other studies. Autologous T cells are then added to the appropriate alveolar macrophage cores, those designated to become “adaptive” granuloma structures, after 48 h of the core’s levitation or 48 h of conventional culture in the case of the traditional cell culture control. Generated structures are then processed individually, in uninfected and infected pairs, for the respective downstream applications desired. These include embedding in tissue-freezing medium for subsequent cryosectioning and staining of the structures for immunofluorescence and confocal microscopy or staining for basic cellular structures like H&E staining or ZN staining for acid-fast bacterial detection (this is exclusively for the 3D structures and cannot be done for the traditional cell culture control cultures). Cells can also be stored for later RNA extractions and subsequent gene expression or RNA sequencing analyses. Supernatants can be stored for cytokine/chemokine production analyses using the Luminex immunoassay platform or similar platforms like ELISA, and cell lysates can be plated to determine CFU counts, thereby evaluating bacterial control.

**Sample collection.** A visual outline of the methods used for this study is shown in Fig. 8.

Bronchoscopies were performed on the study participants by qualified clinicians and nursing staff according to international guidelines (65) and bronchoalveolar lavage done in a segmental bronchus with radiologically suspicious lesions on CXR or computed tomography (CT). The lavage was performed by instilling sterile saline solution at 37°C up to a maximum volume of 300 ml in aliquots of 60 ml at a time, with aspiration between aliquots. Aspirated bronchoalveolar lavage (BAL) fluid was collected in sterile 50-ml polypropylene tubes (Falcon 50-ml sterile conical centrifuge tubes; Corning Inc., NY) and transported on ice to the laboratory. Immediately after bronchoscopy, peripheral blood samples were collected by venipuncture into two 9-ml sodium heparinized (NaHep) vacutainers. Both BAL and peripheral blood samples were processed within 2 h of collection under BSL3 and BSL2 conditions, respectively, owing to the high infectious risk of BAL samples.

**Cell isolations.** BAL cells (BALC) were isolated by centrifugation of BAL fluid (BALF) for 7 min at 300 × *g* (4°C) following sterile filtration through a 70-μm cell strainer (Falcon 70 μm cell strainer; Corning Inc., NY) and successive wash steps with 1× phosphate-buffered saline (PBS). All cells were counted, and the viability was determined using the trypan blue (0.4%; Thermo Fisher Scientific Inc., Waltham, MA) exclusion method. BALC were cultured overnight to isolate adherent cells (the majority of which are alveolar macrophages [AM]) from nonadherent cells (lymphocytes) should the fraction contain lymphocytes exceeding 20% of total cells and then cryopreserved in cryomedium consisting of 10% dimethyl sulfoxide (DMSO; Sigma-Aldrich Co., St. Louis, MO) and 90% fetal bovine serum (FBS; HyClone, GE Healthcare Life Sciences, IL, USA).

Peripheral blood mononuclear cells (PBMC) were isolated from peripheral blood by centrifugation using Ficoll density gradient medium (Histopaque-1077; Sigma Chemical Co., St. Louis, MO). Cells were counted, and the viability was determined using the trypan blue exclusion method. PBMC were then used to isolate autologous CD3^+^ T cells using the MACS (magnetically activated cell sorting) MicroBead isolation technique (Miltenyi Biotec, Cologne, Germany) according to the manufacturer’s instructions. Both the CD3^+^ T cells and the CD3^−^ cell fractions were cryopreserved in cryomedium (described above).

**Thawing of cryopreserved cells.** Cryopreserved cells were thawed in a water bath (37°C) and transferred to 10 ml of warmed complete RPMI 1640 media (supplemented with 1% l-glutamine [Sigma-Aldrich, St. Louis, MO, USA] and 10% FBS). Cells were centrifuged at 300 × *g* for 10 min and washed twice. All cells were counted, and the viability was determined using the trypan blue exclusion method.

**AnaeroPack experiment.** BALC were seeded in a 24-well low-adherence plate at a concentration of 2 × 10^6^ cells per 500 μl complete RPMI. A 2-ml screw-cap tube containing 2 × 10^6^ BALC in 500 μl complete RPMI was cultured separately and acted as the untreated control. A 100 μM concentration of cobalt chloride (CoCl_2_; Sigma-Aldrich, catalogue number C8661-25G) was added to each well to stabilize HIF-1α before the plate was placed in a lock-seal box. Working quickly, the AnaeroPack (Mitsubishi Gas Chemical Co. Inc.) was removed from the protective foil and placed within the lock-seal box next to the culture plate, and the box was quickly sealed shut. The AnaeroPack system is an easy anaerobic atmosphere cultivation method that has no need for either water or catalyst. AnaeroPack creates an environment of <0.1% oxygen and >15% CO_2_. Cells were incubated for 24 h (37°C, 5% CO_2_), after which 125 μl of 16% paraformaldehyde (PFA; ThermoFisher Scientific, catalogue number 28908) was immediately added to the well and untreated control tube (to make a final concentration of 4% PFA) after opening the lock-seal box. The box was resealed, allowing for the cells to fix at room temperature for 20 min. Cells were transferred out of the plate to a 15-ml Falcon tube and centrifuged at 400 × *g* for 10 min. The supernatant was removed, and the cells were resuspended in 1 ml 1× PBS. Cytospins of 2 × 10^5^ BALC were then created for both the treated and untreated control cells on poly-l-lysine-coated microscope slides (Sigma-Aldrich, catalogue number P0425-72EA) to be used for immunofluorescent staining.

**BCG infection.** Cultures of Mycobacterium bovis Bacille Calmette-Guerin (BCG) were grown in Difco Middlebrook 7H9 (BD Pharmingen, San Diego, USA) supplemented with 0.2% glycerol (Sigma-Aldrich), 0.05% Tween 80 (Sigma-Aldrich), and 10% Middlebrook oleic acid albumin dextrose catalase (OADC) enrichment (BD Pharmingen, San Diego, USA). Aliquots of BCG cultures at an optical density at 600 nm (OD_600_) of 0.8 were stored at −80°C in RPMI 1640 medium supplemented with 10% glycerol (Sigma-Aldrich). The number of viable bacteria was assessed by thawing a frozen aliquot and plating serial dilutions onto Middlebrook 7H11 (BD Pharmingen, San Diego, USA) agar plates. The plates were incubated at 37°C (5% CO_2_) for 21 days, and colonies were counted manually thereafter to determine the number of viable bacteria.

AM were thawed and rested at 37°C (5% CO_2_) for 18 h in complete RPMI 1640 supplemented with 1% l-glutamine (Sigma-Aldrich, St. Louis, MO, USA), 10% FBS, and an antimycotic antibiotic containing 10,000 U/ml penicillin, 10,000 μg/ml streptomycin, and 25 μg/ml amphotericin B (Fungizone) (PSF; Lonza, Walkersville, MD, USA). The following day, AM were washed in complete RPMI lacking PSF, centrifuged at 300 × *g* for 10 min, and then cultured at a density of 4 × 10^5^ cells/well in a 24-well low-adherence culture plate (Greiner Bio-One, NC, USA). Both “uninfected” and “infected” wells were seeded for comparison between infection states. Infected wells were infected with BCG (Pasteur strain, MOI 1) and incubated for 4 h. Extracellular bacteria were removed by incubating cells (37°C, 5% CO_2_) with complete RPMI supplemented with PSF for 1 h, followed by successive washes with complete RPMI lacking PSF.

**3D spheroid granuloma construction.** Magnetic cell levitation and bioprinting are recently developed methods used to generate 3D tumor spheroids (58, 59). We have implemented this system to create incipient, innate-style and mature, classic (adaptive)-style 3D lung granuloma types resembling TB granulomas, which will henceforth be referred to as innate and adaptive granulomas, respectively. It should be noted that all incubation periods mentioned in this methods section are carried out in an incubator (37°C, 5% CO_2_) and that paired BALC and PBMC samples were used to build each spheroid.

**FIG 9:**
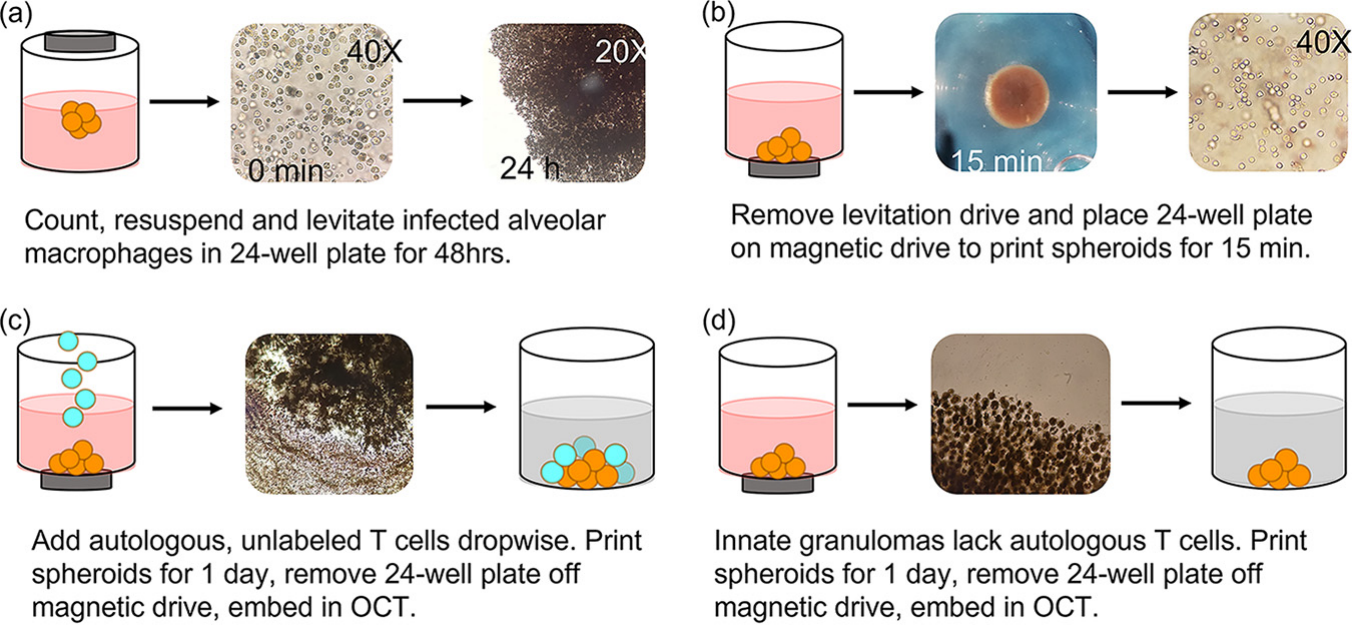
3D innate and adaptive granuloma construction using the n3D Biosciences Inc. magnetic levitation and printing drives. (a) The development of the granuloma core is accomplished through the levitation of NanoShuttle-labeled alveolar macrophages for 48 h. (b) The magnetic levitation drive is removed after 48 h and immediately replaced by the magnetic printing drive below the 24-well culture plate, which ensures that the 3D structure remains intact. (c) Autologous CD3^+^ T cells, not labeled with NanoShuttle, are then carefully added to the alveolar macrophage core and allowed to migrate via chemotactic gradients to the core to create the mature, adaptive granuloma. (d) Innate granulomas do not have the autologous CD3^+^ T cells added to the core but are rather left with the printing drive secured below the culture plate for the remainder of the experiment.

In brief, BCG-uninfected and -infected AM were treated overnight with biocompatible NanoShuttle (made from gold, iron oxide, and poly-l-lysine; n3D Biosciences Inc., Greiner Bio-One) at a concentration of 100 μl per 1 × 10^6^ cells in low-adherence plates at a maximum volume of 300 μl per well (15, 60). The following day, the NanoShuttle-labeled AM were levitated using the magnetic levitating drive (n3D Biosciences Inc., Greiner Bio-One) for 48 h to cluster and assemble the spheroid core (Fig. 9a). This core consisting of only AM is considered the innate granuloma. Concurrently, autologous CD3^+^ T cells were thawed, counted, and rested overnight in complete RPMI in a separate 24-well culture plate. Adaptive granulomas were created 48 h postconstruction of the spheroid core, through addition of autologous T cells (6 × 10^5^/well) at a ratio of 40:60 (AM:T cells). We previously conducted a titration of cell ratios (data not shown) and selected a macrophage to lymphocyte ratio that macroscopically resembles a TB lung granuloma considered to harbor a median bacterial burden (42). This was achieved by carefully removing the levitating drive from the culture plate (Fig. 9b) and replacing it with the magnetic printing drive (Fig. 9c). Chemokine production by the AM core allows for T cell migration to the spheroid core, forming an outer lymphocytic cuff, resembling the complex *in vivo*, organized mature classic human TB granulomas. The adaptive granuloma was then incubated for 24 h, after which the printing drive was removed and each spheroid granuloma was dedicated to various downstream processes. This resulted in a total culture period of 5 days postinfection and was decided upon based on previous experiments which looked at the determination of adequate cell viability, the ability to conduct single-cell analyses, the stability of the structure, and postculture cell yield. For the innate granulomas, no autologous T cells were added, but the core was incubated for the same period as the adaptive granulomas (Fig. 9d). Nanospheres subsequently detach from the cells, allowing unsupported growth as the structure starts to mimic extracellular matrix (ECM) conditions. This model thus allows for the addition, enrichment, or depletion of specific innate or adaptive immune cells or immune mediators to interrogate their potential contribution to protective or nonprotective granulomatous responses.

**Traditional cell culture control construction.** As a control culture condition, traditional cell cultures were constructed in tandem with the 3D spheroid granuloma construction in a separate 24-well low-adherence culture plate for each participant. The exact same ratios of AM to T cells were used for these control cultures, the only difference being that no NanoShuttle was added to the AM, and subsequently no levitation or printing drives were added to the culture plate to replicate cellular distributions under normal *in vitro* culture conditions. Both uninfected and infected control cultures were set up for both innate and adaptive granuloma types.

### 3D spheroid granuloma embedding and cryosectioning.

A pair of uninfected and infected spheroid granulomas had their supernatant carefully removed and stored in separate 0.2-ml Eppendorf tubes at −80°C for later cytokine response interrogation using Luminex immunoassays. The structures were then washed twice with 300 μl complete RPMI, making sure to not disrupt the structures, and subsequently fixed with 300 μl 4% paraformaldehyde (PFA; ThermoFisher Scientific, catalogue number 28908) for 30 min in the dark. Fixed structures were then embedded in tissue-freezing medium OCT (Tissue-Tek; USA) and stored at −80°C for later cryosectioning. Cryosections were made using a −20°C Cryostat (Leica Biosystems), with sections being cut 7 μm thick and mounted onto poly-l-lysine-coated microscope slides (Sigma-Aldrich, catalogue number P0425-72EA). The cryosections were numbered in order of cutting to allow for the “position” within the structure to be inferred and picked for specific staining. Mounted sections were stored at −20°C until immunohistochemistry staining could be performed. Sections were reserved for immunofluorescent staining (sections from the center of the 3D granuloma structure), H&E staining (a section from near the center), and ZN staining (a section from near the center). The processing described for immunofluorescent staining, immunofluorescent microscopy, H&E staining, Ziehl-Neelsen staining, and light microscopy was not done for the traditional cell culture conditions.

**Staining and microscopy. (i) Immunofluorescent staining.** All staining was done in a humidified chamber. Fixed granuloma sections (stored at −20°C) were rehydrated in 1× PBS for 10 min followed by two washes of 1× PBS for 5 min. Sections were blocked with 1% bovine serum albumin (BSA; Sigma-Aldrich, catalogue number A7030) in PBS containing 0.1 M glycine for 1 h at room temperature, after which the sections were incubated with corresponding primary antibodies diluted in 1% BSA in PBS overnight at 4°C in the dark. Antibody concentrations were as follows: alveolar macrophage membranes were stained using CD206 PE-CF594 (mouse anti-human CD206, BD Biosciences, catalogue number 564063) at a concentration of 4 μl in a 50 μl final staining volume per section, and the universal T cell surface marker CD3 V450 (mouse anti-human CD3, BD Biosciences, catalogue number 560365) was used at a concentration of 5 μl in a 50-μl final staining volume per section. To assess necrosis, the expression of the necrotic marker HMGB1 was assessed using HMGB1 Alexa-Fluor (AF) 647 (mouse anti-human, BioLegend, catalogue number 651408) at a concentration of 20 μg/ml. HMGB1-stained sections were counterstained with the nuclear stain, Hoechst (Life Technologies, catalogue number H3570), at a concentration of 1 μg/ml. Following overnight incubation, sections were washed three times in 1× PBS for 5 min, allowed to dry slightly before being mounted with Dako mounting medium (Agilent Technologies), and allowed to air dry overnight in the dark at room temperature. Slides were stored at 4°C in the dark until imaging. Unstained and single stained controls were also prepared for each experiment to assess background and signal specificity in each channel.

Immunofluorescent staining for cytospin slides (see “AnaeroPack Experiment” above) was performed as described above, with antibody concentrations as follows: BALC were stained with Hoechst (Life Technologies, catalogue number H3570) at a concentration of 1 μg/ml, the hypoxia marker HIF-1α AF488 (mouse anti-human, BioLegend, catalogue number 359708) was used at a concentration of 2.5 μg/ml final staining volume per section, and the necrosis marker HMGB1 AF647 (mouse anti-human, BioLegend, catalogue number 651408) was used at a concentration of 20 μg/ml final staining volume per section.

**(ii) Immunofluorescent microscopy.** Images were obtained using a Carl Zeiss LSM 880 Airyscan with Fast Airyscan Module confocal microscope (Plan-Apochromat ×63/1.40 oil DIC UV-VIS-IR M27 lens objective), and the images were acquired using the ZEN software (Carl Zeiss). Acquisition settings for imaging were identically set for all sections. Red channel: excitation wavelength (561 nm), emission wavelength (659 nm), detection wavelength (585 to 733 nm), pinhole (1.42 AU), frame scan mode, detector gain (640); blue channel: excitation wavelength (405 nm), emission wavelength (432 nm), detection wavelength (410 to 455 nm), pinhole (2.17 AU), frame scan mode, detector gain (724). Eighteen by eighteen tile scans were acquired and stitched together.

**(iii) H&E staining.** One section from both uninfected and infected granuloma structures, not reserved for immunofluorescent staining and control slides, was stained using the Lillie Mayer H&E method (61) to visualize the overall compartmentalization of the various cell types added to the 3D structure without immunofluorescent stains. Briefly, the designated sections were removed from storage and allowed to reach room temperature while placed in a slanted position and rehydrated in dH_2_O. Sections were then stained with alum hematoxylin for 4 min, rinsed with tap water, and differentiated with 0.3% acid-alcohol for 2 s. Sections were rinsed again with tap water, then rinsed in Scott’s tap water substitute, and rinsed again with tap water. Finally, sections were stained with eosin for 2 min, dehydrated, and cleared, and coverslips were mounted using 1 drop of VectaMount (Vector Laboratories, CA, USA).

**(iv) Ziehl-Neelsen (ZN) staining.** One section from both uninfected and infected granuloma structures, not reserved for immunofluorescent staining and control slides, was stained using the ZN method to identify the localization of acid-fast bacteria within the 3D structure. Briefly, the designated sections were removed from storage and allowed to reach room temperature while placed in a slanted position. Sections were then flooded with carbol fuchsin and heated gently with a flame until a vapor was emitted. Sections were immersed for 5 min, rinsed with dH_2_O, flooded with acid-alcohol, and allowed to stand for 2 min. Following this, sections were rinsed with dH_2_O, counterstained with methylene blue, allowed to stand for 2 min, and finally rinsed and allowed to dry. Dry sections were mounted with coverslips using 1 drop of VectaMount.

**(v) Light microscopy.** H&E-stained sections and ZN-stained sections were visualized using the ZEISS Axio Observer microscope and fitted with an Axiocam MRc 195 microscope camera, and the images were acquired using the ZENlite imaging software (blue edition, version 1.1.1.0).

**Flow cytometry.** An uninfected and infected granuloma pair was mechanically dissociated (by gentle pipetting) into single cells and transferred to respective sterile 15 ml Falcon tube. Cells were then centrifuged at 300 × *g* for 10 min, resuspended in 5 ml complete RPMI, and washed. Washed cells were then resuspended in 300 μl complete RPMI and transferred to a sterile, 24-well culture plate (untreated), after which cells were incubated for 48 h to allow for adherence of the AM. Following incubation, the nonadherent fraction was separated from the adherent cells and the wells were washed twice. These cells were immediately used for the flow cytometric investigation of the phenotypic and functional characteristics of the nonadherent cellular fraction, namely, T cells. The same procedure was followed for an uninfected and infected traditional cell culture control pair. The adherent fraction was not analyzed by flow cytometry, due to the high levels of autofluorescence. To demonstrate this, 1 × 10^6^ total BALC and PBMC were transferred to two separate 5-ml Falcon tubes and centrifuged at 250 × *g* for 5 min. Both fractions were kept unstained and acquired on the BD FACS Canto II, with gates being set using a predefined fluorescence-minus-one (FMO) from a stained PBMC sample with the channels CD3 PE-Cy7, CD14 Pacific Blue, and anti-HLA-DR APC.

Nonadherent fractions were analyzed by flow cytometry using the BD FACS Canto II and were stained to define their basic phenotypic profiles. Fractions were stained using anti-CD3 PE-Cy7 (BD Biosciences, catalogue number 563423), anti-CD14 Pacific Blue (BD Biosciences, catalogue number 558121), anti-CD16 PerCP-Cy5.5 (BD Biosciences, catalogue number 565421), and anti-CD56 BV510 (BD Biosciences, catalogue number 563041). Staining was performed for 30 min in the dark at room temperature in a 50-μl staining volume. Titrations, compensation, and FMO for each antibody were performed prior to analysis at the BD Stellenbosch University flow cytometry unit on Tygerberg Campus. Data were analyzed using the third-party FlowJo software version 10.0.8 and the 3D granuloma structure data compared to that obtained for the traditional cell culture control.

**RNA sequencing. (i) RNA extractions.** An uninfected and infected granuloma pair was mechanically dissociated (by gentle pipetting) into single cells and transferred to a sterile 2-ml screw-cap tube. Cells were then centrifuged at 300 × *g* for 10 min and vigorously resuspended in 350 μl RLT buffer (Qiagen, Germany) by vortexing for 30 s. Cells were stored at −80°C for batched RNA extractions to perform gene expression or RNA-Seq analysis. On the day of batched RNA extractions, samples were removed from storage and allowed to thaw at room temperature. RNA was extracted using the Qiagen RNeasy minikit according to the manufacturer’s instructions. Following isolation, 1 μl of each sample was used to check RNA integrity using a Nanodrop spectrophotometer (Thermo Fisher Scientific, MA, USA), with all samples having an *A*_260_/*A*_280_ ratio of above 1.8. The remaining RNA was stored until RNA sequencing could be performed. The same procedure was followed for an uninfected and infected traditional cell culture control pair.

**(ii) Library preparation.** Total RNA was extracted from 11 samples; however, 2 samples did not meet the total RNA (200 ng) requirements for further processing. Total RNA was subjected to DNase treatment and magnetic bead-based mRNA enrichment using the Dynabeads mRNA purification kit (Invitrogen, Thermo Fisher Scientific, Waltham, MA, USA), according to the protocol described in the *MGIEasy RNA Library Prep Set User Manual*, prior to proceeding with library construction. Library preparation was performed with the entire component of mRNA for each sample using the MGIEasy RNA library prep kit (MGI, Shenzen, China), according to the manufacturer’s instructions.

**(iii) Sequencing.** Massively parallel sequencing was performed at the South African Medical Research Council (SAMRC)/Beijing Genomics Institute (BGI) Genomics Centre using DNA nanoball-based technology on the MGISEQ-2000 using the appropriate reagents supplied in the MGISeq-2000RS high-throughput sequencing kit. A paired-end sequencing strategy was employed with a read length of 100 bp (PE100). Sample libraries were loaded onto MGISEQ-2000 FCL flow cells with the MGILD-200 automatic loader, and 18 FASTQ files were generated (9 forward and 9 reverse read files).

**(iv) Analysis.** Raw FASTQ files were assessed using FastQC (version 0.11.5). Reads with quality scores lower than 20 and length below 30 bp were all trimmed. The resulting high-quality sequences were subsequently used in MultiQC, a module contained in Python (version 3.6.3), to aggregate and summarize the results from multiple FastQC reports into a single HTML report. Raw FASTQ files were then imported, annotated (human GRCh38.p13 data set from https://www.ncbi.nlm.nih.gov/assembly/GCF_000001405.39/), filtered (counts per million [CPM] cutoff method), normalized, and analyzed using statistical software based in R (version 3.6.3). Differentially regulated transcripts were functionally annotated to gain an overview of biological pathway regulation. Briefly, GO terms enrichment analysis was conducted on the ensemble gene IDs using the Database for Annotation, Visualization, and Integrated Discovery (DAVID) version 6.8 (62, 63), while the REVIGO resource was used to summarize and visualize the most enriched GO terms (64).

**Luminex immunoassay.** As mentioned previously in Materials and Methods, a pair of uninfected and infected granulomas had their supernatant removed and stored in separate 0.2-ml Eppendorf tubes at −80°C for later cytokine response interrogation using the Luminex immunoassay platform (Luminex, Bio Rad Laboratories, Hercules, CA, USA). Supernatants were collected and stored from structures throughout the culture period, beginning from 1 day to 4 days postinfection, at the beginning of each day’s processing. The same procedure was followed for an uninfected and infected traditional cell culture control pair. A one-plex kit was used to measure the production of interleukin 22 (IL-22; R&D Systems, LXSAHM-01), and a four-plex was used to measure the production of IL-2, IL-10, gamma interferon (IFN-γ), and tumor necrosis factor alpha (TNF-α) (R&D Systems, LXSAHM-04). Briefly, samples were removed from storage and allowed to reach room temperature 1 h before the assay was begun. Samples were then vortexed and prepared for the assay according to the manufacturer’s instructions. Samples were not diluted as recommended by the manufacturer but run neat due to the small number of cells used in culture and the restrictive lower limit of detection observed for Luminex immunoassays. Samples were run on the Luminex MAGPIX system. The beads from each sample were acquired individually and analyzed using the Bio-Plex Manager software version 6.1 according to recommended settings. Instrument settings were adjusted to ensure 50 bead events per region, with sample size set to 50 μl for both kits.

**CFU determination.** The supernatant from the remaining uninfected and infected granuloma pair was carefully removed, and the cells were mechanically dissociated (by gentle pipetting) into single cells after 300 μl complete RPMI was added. Cells were then transferred to a sterile 15-ml Falcon tube, centrifuged at 300 × *g* for 10 min, resuspended in 200 μl dH_2_O to lyse the cells, and vortexed. Serial dilutions were prepared using this cell lysate and PBS-Tween 80. The neat cell lysate and serial dilutions were then plated out in duplicate on Middlebrook 7H11 agar plates (BD Biosciences) for manual CFU determination after 21 days. The same procedure was followed for an uninfected and infected traditional cell culture control pair.

### Statistical analysis.

Statistical analyses were performed using GraphPadPrism version 8 (GraphPad Software, San Diego, CA). A *P* value of less than 0.05 was considered significant. Tests for normality could not be performed due to sample sizes being too small in this pilot study; data were, therefore, treated as nonparametric throughout. Where two groups were compared, the Mann-Whitney *t* test was used. The Kruskal-Wallis test was performed using Dunn’s posttest to correct for multiple comparisons where three or more groups were compared.
