# Supplementary figures and images for: Establishment of a Patient-Derived, Magnetic Levitation-Based, Three-Dimensional Spheroid Granuloma Model for Human Tuberculosis
Source: mSphere. 2021 Jul 21;6(4):e00552-21. doi: 10.1128/mSphere.00552-21 (PMC8386456; doi:10.1128/mSphere.00552-21)

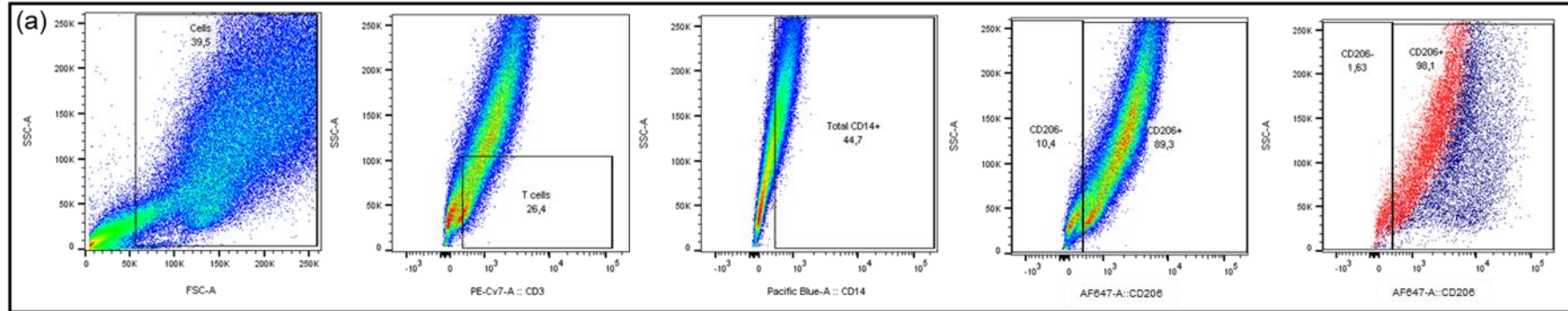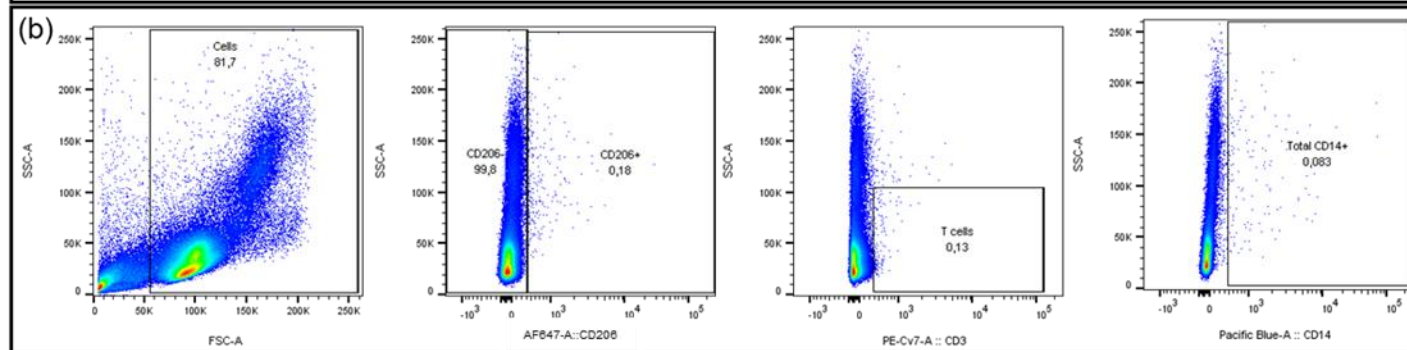

| Sample Name   |
|---------------|
| Unstained     |
| CD206 (AF647) |

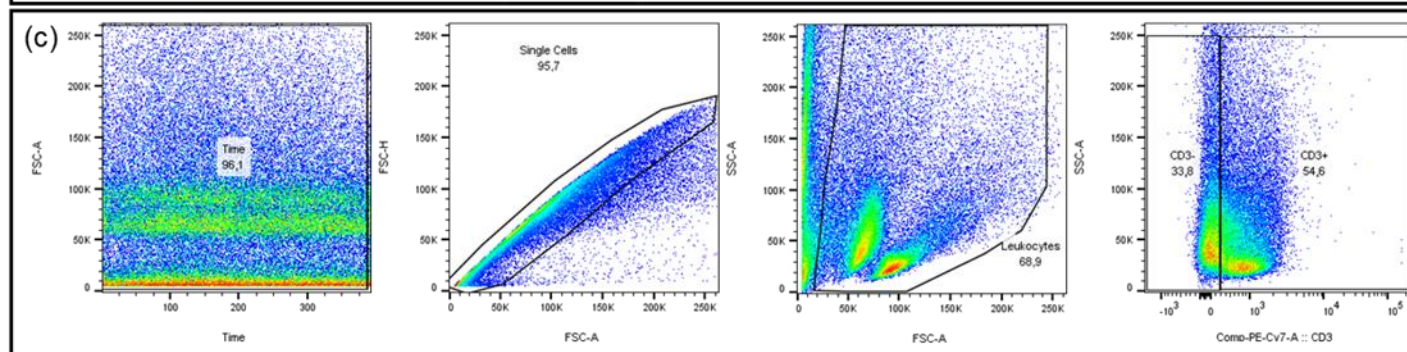

Supplement: FIG S1 [file msphere.00552-21-sf001.pdf]

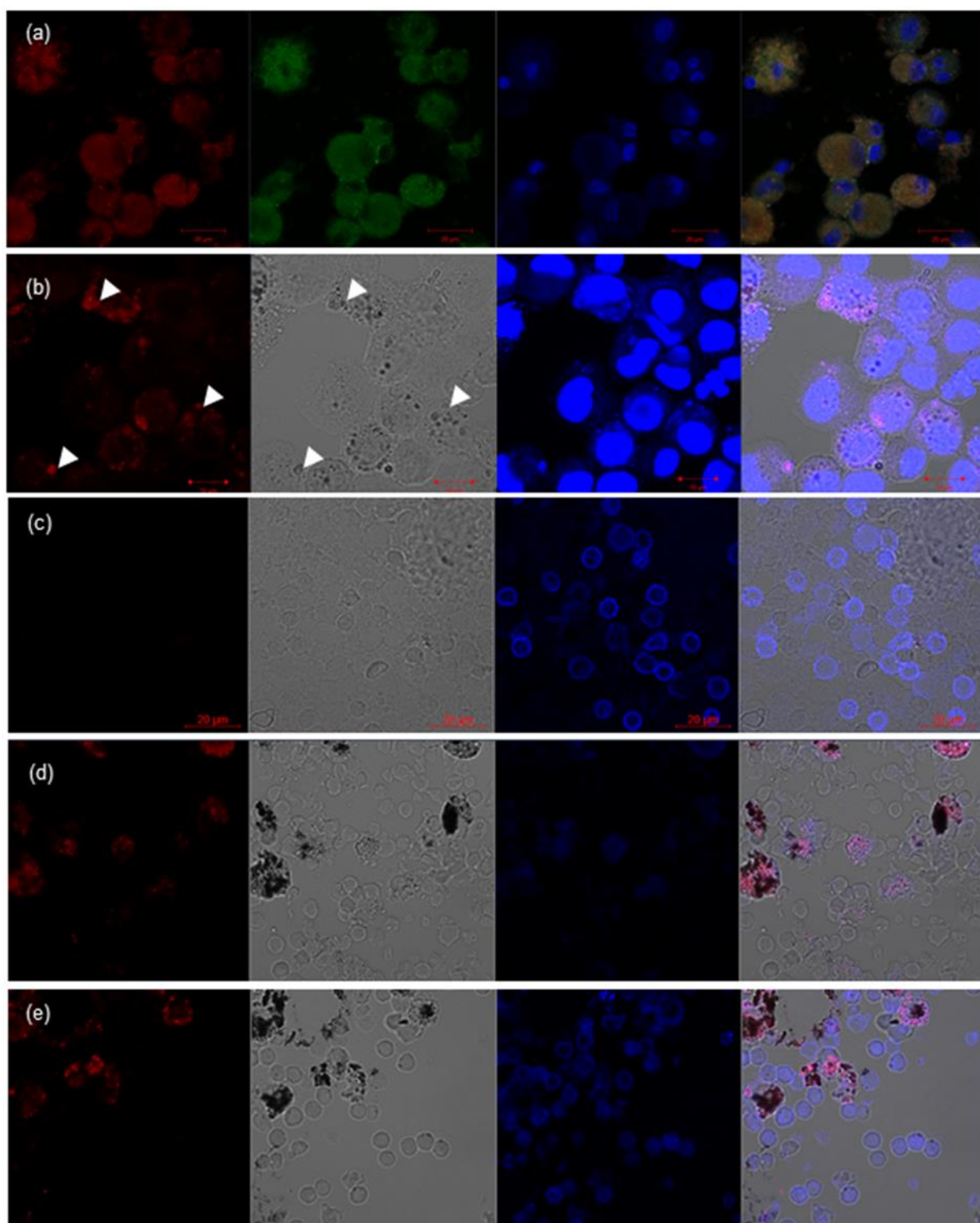

Supplement: FIG S2 [file msphere.00552-21-sf002.pdf]

Forward Reads

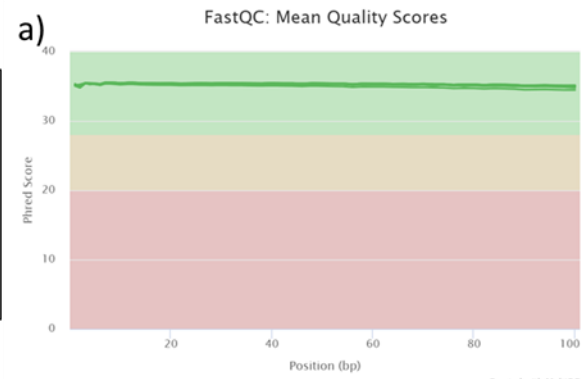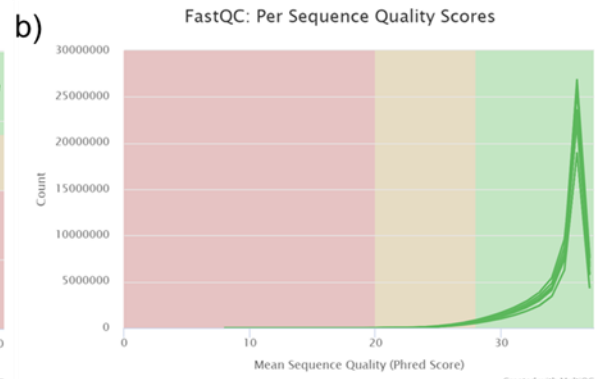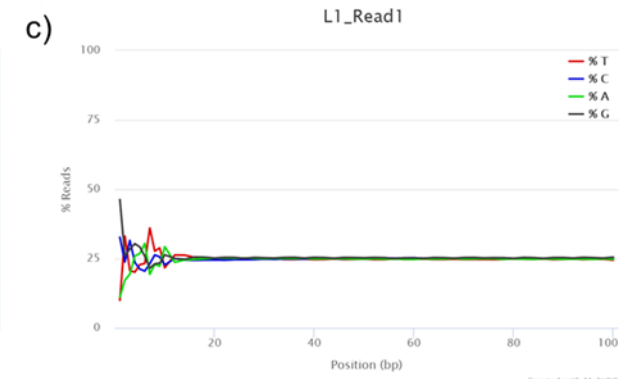

Reverse Reads

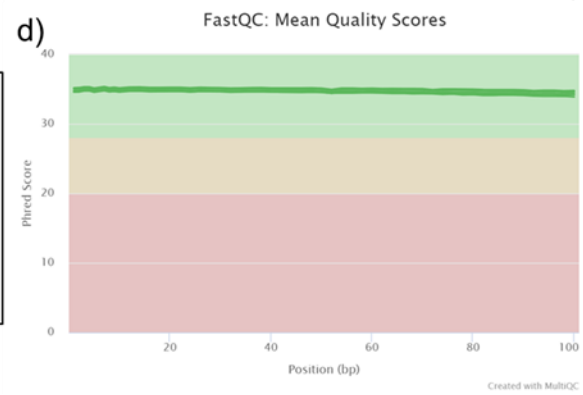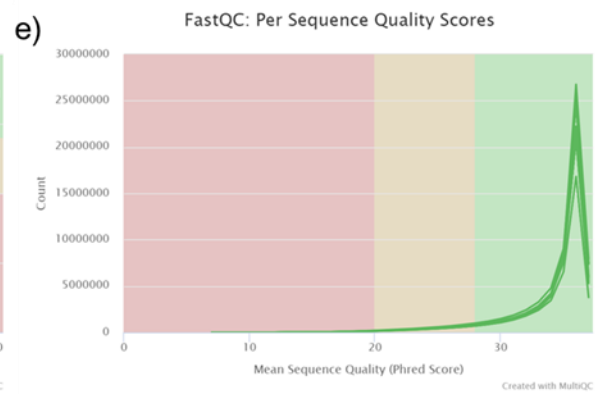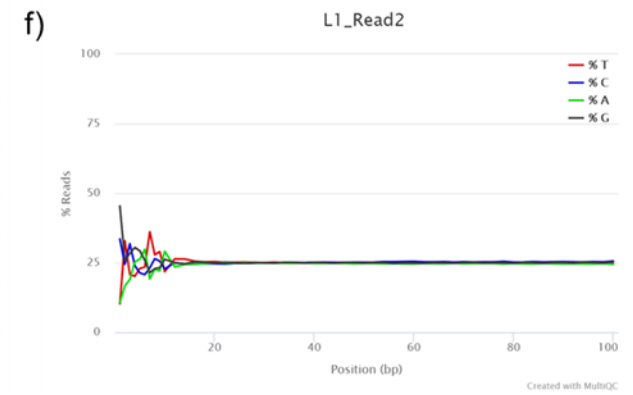

Supplement: FIG S3 [file msphere.00552-21-sf003.pdf]

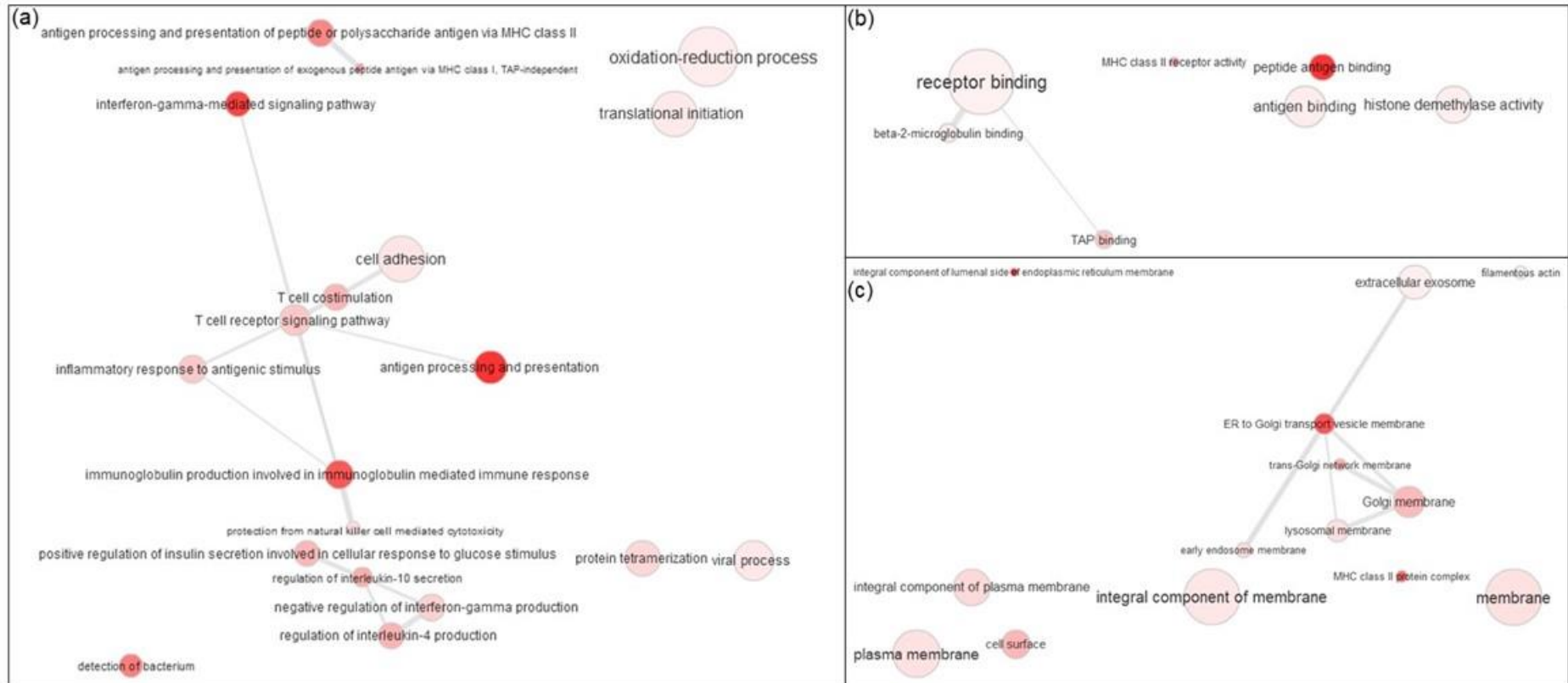

Supplement: FIG S4 [file msphere.00552-21-sf004.pdf]
