# Supplementary material for: Establishment of a Patient-Derived, Magnetic Levitation-Based, Three-Dimensional Spheroid Granuloma Model for Human Tuberculosis
Source: mSphere. 2021 Jul 21;6(4):e00552-21. doi: 10.1128/mSphere.00552-21 (PMC8386456; doi:10.1128/mSphere.00552-21)
Supplement: TABLE S1 [file msphere.00552-21-st001.pdf]

	D.P.C.I.	Uninfected Chronic Granuloma			Infected Chronic Granuloma			Uninfected Innate Granuloma			Infected Innate Granuloma			Infected Monolayer			T cells		
		TBH7760	TBH6999	TBH1252	TBH7760	TBH6999	TBH1252	TBH7760	TBH6999	TBH1252	TBH7760	TBH6999	TBH1252	TBH7760	TBH6999	TBH1252	TBH7760	TBH6999	TBH1252
IL-10 (pg/ml)	1																		
	2	2,41	0,25	114,92	16,5	9,87	554,02	2,41	0,25	114,92	16,5	9,87	554,02	12,53	3,56	563,94			
	3	0,49	0,79	178,46	11,64	17,11	522,33	0,49	0,79	178,46	11,64	17,11	522,33	1,43	18,52	531,87			
	4	0,2	0,66	94,2	6,08	18,86	281,61	0,2	0,66	94,2	6,08	18,86	281,61	0,53	14,38	263,46	4,62	24,41	16,02
	5	24,61	7,22	39,53	7,88	36,07	186,5	0,96	1,43		1,96	25,03		0,96	34,34	102,11			
IFN-g (pg/ml)	1																		
	2	8,5	8,5	16478,88	9,04	236,23	11036,85	8,5	8,5	16478,88	9,04	236,23	11036,85	16,7	202,24	23130,46			
	3	8,5	8,5	5530,39	25,55	178,9	2413,32	8,5	8,5	5530,39	25,55	178,9	2413,32	37,2	66,48	2888,29			
	4	8,5	8,5	2726,5	32,29	110,46	1330,11	8,5	8,5	2726,5	32,29	110,46	1330,11	8,5	46,74	1314,81	163,52	1332,66	804,73
	5	10311,46	176,35	3858	6088,76	352,44	1233,12	8,5	8,5		37,2	49,85		1206,55	369,71	3554,87			
TNF-a (pg/ml)	1																		
	2	29,44	8,16	28108,35	671,09	19376,15	24436	29,44	8,16	28108,35	671,09	19376,15	24436	499,3	15305,08	29045,11			
	3	2,87	31,01	22833,81	418,3	10559,45	23455,02	2,87	31,01	22833,81	418,3	10559,45	23455,02	1976,06	11729,48	25537,76			
	4	2,87	23,34	23815,8	77,99	1375,83	15192,27	2,87	23,34	23815,8	77,99	1375,83	15192,27	34,36	3623,98	21760,67	318,69	452,83	451,12
	5	2193,36	94,94	20385,24	1202,57	622,88	10885,04	11,28	19,68		66,68	271,68		5169,96	3050,25	15848,48			
IL-2 (pg/ml)	1																		
	2	1,34	1,94	3718,77	7,24	68,47	2688,89	1,34	1,94	3718,77	7,24	68,47	2688,89	8,4	48,75	3378,7			
	3	0,72	0,72	6,66	2,54	5,49	5,49	0,72	0,72	6,66	2,54	5,49	5,49	6,66	3,73	5,49			
	4	0,5	1,34	6,37	2,54	1,94	4,32	0,5	1,34	6,37	2,54	1,94	4,32	0,72	2,54	4,32	21,5	3,14	11,28
	5	127,36	6,08	13,57	147,16	6,08	4,91	1,94	1,94		1,94	1,94		716,35	8,4	11,28			
IL-22 (pg/ml)	1																		
	2	10,37	2,5	12,12	10,37	3,63	18,47	10,37	2,5	12,12	10,37	3,63	18,47	12,54	2,5	31,6			
	3	10,37	2,5	2,5	5,43	7,54	2,5	10,37	2,5	2,5	5,43	7,54	2,5	11,25	5,43	2,5			
	4	14,19	11,25	2,5	13,79	8,52	2,5	14,19	11,25	2,5	13,79	8,52	2,5	7,54	7,54	2,5	4,26	3,63	2,5
	5	71,12	2,5	2,5	57,06	2,5	2,5	10,37	5,43		12,96	2,94		36,69	2,5	2,5			

Abbreviations: D.P.C.I. – Days Post Culture Initiation; IL – interleukin; IFN-g – Interferon gamma; TNF-a – Tumor Necrosis Factor alpha
